# IGF2BP2-meidated m^6^A modification of CSF2 reprograms MSC to promote gastric cancer progression

**DOI:** 10.1038/s41419-023-06163-7

**Published:** 2023-10-21

**Authors:** Runbi Ji, Chenxi Wu, Jun Yao, Jiajin Xu, Jiang Lin, Hongbing Gu, Min Fu, Xiaoxin Zhang, Yongkang Li, Xu Zhang

**Affiliations:** 1 Department of Gastroenterology, Institute of Digestive Diseases, The Affiliated People’s Hospital of Jiangsu University, Zhenjiang, 212002 Jiangsu China; 2 Jiangsu Key Laboratory of Medical Science and Laboratory Medicine, School of Medicine, Jiangsu University, Zhenjiang, 212013 Jiangsu China; 3 Department of Clinical Laboratory, The Affiliated People’s Hospital of Jiangsu University, Zhenjiang, 212002 Jiangsu China; 4 Department of Central Laboratory, The Affiliated People’s Hospital of Jiangsu University, Zhenjiang, 212002 Jiangsu China

**Keywords:** Mesenchymal stem cells, Reprogramming

## Abstract

The interaction between tumor cells and stromal cells within the tumor microenvironment plays a critical role in cancer progression. Mesenchymal stem cells (MSCs) are important tumor stromal cells that exhibit pro-oncogenic activities when reprogrammed by the tumor. However, the precise mechanisms underlying MSC reprogramming in gastric cancer remain not well understood. QRT-PCR, western blot, and immunohistochemistry were used to examine gene and protein expression levels. In vitro and in vivo experiments were conducted to assess the biological functions of gastric cancer cells. RNA-sequencing, RNA immunoprecipitation (RIP), and meRIP assays were performed to investigate underlying molecular mechanisms. We found a significant increase in the expression and N6-methyladenosine (m^6^A) modification levels of colony-stimulating factor 2 (CSF2) in gastric cancer MSCs. CSF2 gene overexpression induced the reprogramming of normal MSCs into cancer-promoting MSCs, thereby enhancing the proliferation, migration, and drug resistance of gastric cancer cells through the secretion of various pro-inflammatory factors. Additionally, we demonstrated that the m^6^A reader IGF2BP2 bound to and stabilized CSF2 mRNA in gastric cancer MSCs. Notably, overexpression of IGF2BP2 mimicked the effect of CSF2 on MSCs, promoting gastric cancer progression. Finally, we unveiled that CSF2 induced the ubiquitination of Notch1 to reprogram MSCs. Our study highlights a critical role of IGF2BP2-mediated m^6^A modification of CSF2 in reprogramming MSCs, which presents a promising therapeutic target for gastric cancer.

## Introduction

Gastric cancer ranks as the fifth most prevalent cancer globally [1], and the overall survival rate of advanced disease remains low [2], which may be attributed to the heterogeneous and intricate nature of the tumor microenvironment [3]. Tumor microenvironment reprogramming contributes to the progression and metastasis of gastric cancer [4]. Targeting tumor microenvironment cells offers a novel approach to enhance the prognosis of patients with gastric cancer.

Mesenchymal stem cells (MSCs) play a crucial role in orchestrating the tumor microenvironment and regulating many aspects of tumor cells, including cell proliferation, metastasis, angiogenesis, and immune evasion [5–7]. Notably, previous studies demonstrate that MSCs have remarkable plasticity of and exhibit specific reprogramming within distinct microenvironments through various mechanisms [8, 9]. Consequently, reprogrammed MSCs induce epithelial-mesenchymal transition (EMT), activate cell proliferation, sustain tumor stem cell niche, and modulate tumor cell metabolism [10]. We have previously revealed the presence of MSCs with tumor-promoting capabilities in gastric cancer tissues [11]. Numerous external and internal factors, such as gastric cancer-derived exosomes [12], specific anti-cancer medications [13], and dysregulated miRNA expression [14], could reprogram MSCs into a tumor-promoting phenotype, contributing to the development and progression of gastric cancer.

The mechanism underlying MSCs reprogramming in cancer remains not well understood. N6-methyladenosine (m^6^A) modification represents the most prevalent form of mRNA methylation in mammalian cells. In gastric cancer, the elevated RNA m^6^A level has been linked to a poor prognosis [15]. The m^6^A writer METTL3 targets HDGF [15] and ZMYM1 [16], promoting the growth and metastasis of gastric cancer cells. Furthermore, m^6^A modification plays a critical role in regulating the phenotype of stem cells. Hematopoietic stem cells, for instance, exhibit significant m^6^A modification in 2073 genes [17]. Notably, m^6^A modification governs the differentiation of normal bone marrow stem cells [18–20] and sustains the self-renewal potential of breast cancer stem cells [21]. However, the precise role of m^6^A modification in MSCs within the tumor microenvironment remains unclear. We hypothesized that the altered RNA m^6^A modification may regulate the phenotype and functionality of MSCs, consequently promoting gastric cancer progression.

In this study, we identified an elevation of colony stimulating factor 2 (CSF2) in gastric cancer tissue-derived mesenchymal stem cells (GC-MSCs) through m^6^A-dependent mechanism. CSF2 upregulation induced the transition of MSCs towards a cancer-promoting phenotype, thereby facilitating the proliferation, migration, and invasion of gastric cancer cells. The m^6^A reader protein IGF2BP2 bound to and augmented the stability of CSF2 mRNA. Additionally, CSF2 negatively regulated the Notch signaling pathway through the induction of Notch1 ubiquitination. Our study elucidates a novel mechanism by which MSC phenotype and function is regulated by m^6^A modification in gastric cancer. This finding represents an additional layer of modulation for MSC reprogramming within the tumor microenvironment and provides a novel strategy for the treatment of gastric cancer.

## Results

### CSF2 expression and m^6^A modification increase in tumor-conditioned MSCs

**Fig. 1:**
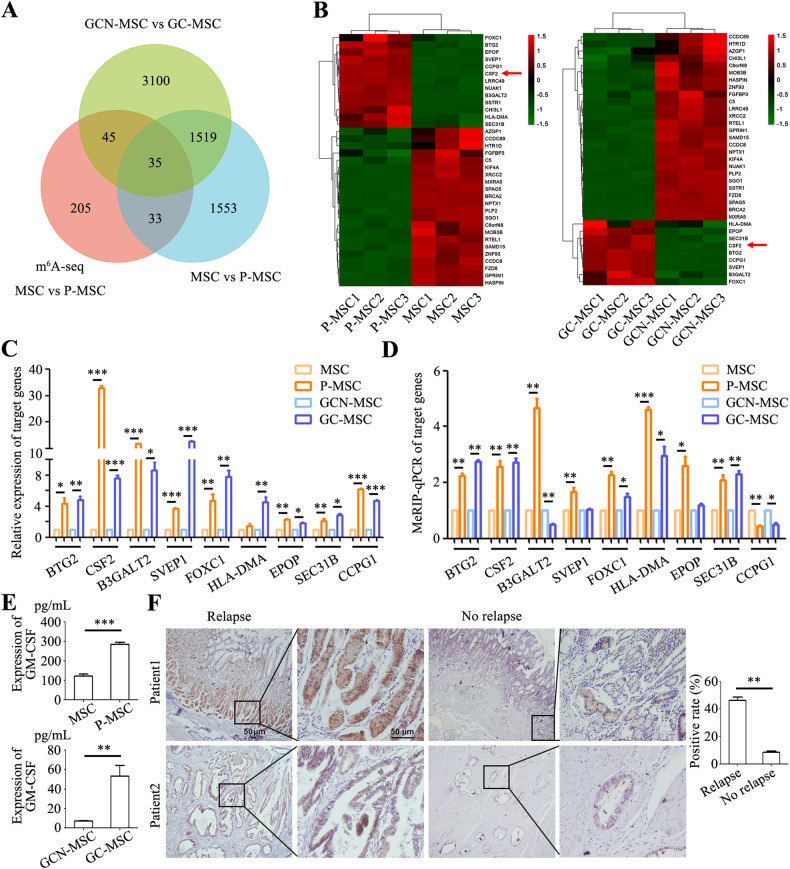
CSF2 expression and m^6^A modification increase during MSC reprogramming. **A** Venn diagram depicting the overlapped genes between RNA-seq and m^6^A-seq data. **B** Cluster analysis of 35 genes showing differential expression and m^6^A modification. **C** QRT-PCR analyses of the expression levels of nine upregulated genes. **D** MeRIP-qPCR analyses of the m^6^A levels of nine upregulated genes. **E** ELISA analyses of CSF2 protein (GM-CSF) expression in the supernatants from MSCs, P-MSCs, GCN-MSCs and GC-MSCs. **F** Immunohistochemical analyses of CSF2 expression in gastric cancer tissues. Magnification: ×40 and ×200. Scale bar = 50 μm. *P < 0.05; **P < 0.01; ***P < 0.001.

We first examined the changes in the transcriptome of MSCs from distinct sources, including GC-MSCs (gastric cancer tissues derived MSCs) and GCN-MSCs (non-cancerous tissues derived MSCs), normal MSCs and P-MSCs (MSCs pre-treated with conditioned medium from gastric cancer cells). The immunophenotype and multi-differentiation abilities of MSCs were characterized by FACS analyses (positive for CD29, CD44H, and CD90 and negative for CD45) and induced differentiation assays (alkaline phosphatase staining for osteogenic induction and Oil Red O staining for adipogenic induction) (Fig. S1A, B). The results of microarray analyses showed that a total of 4699 genes displayed altered expression in GC-MSCs compared to GCN-MSCs and 3140 genes had changed levels in P-MSCs compared to normal MSCs (Fig. 1A). We then performed RNA sequencing for m^6^A modification (m^6^A-seq) in normal MSCs and P-MSCs. The results of m^6^A-seq showed that 318 genes had significant alteration of m^6^A levels in P-MSCs compared to normal MSCs, of which 35 genes exhibited changes in both expression and RNA m^6^A levels (Fig. 1A). Cluster analysis of these 35 genes identified 9 genes that were concurrently up-regulated in both GC-MSCs and P-MSCs (Fig. 1B). To validate RNA-seq and m^6^A-seq data, we examined the expression levels and m^6^A peak levels of the overlapped 9 genes by qRT-PCR and meRIP-qPCR and found that CSF2 expression in P-MSCs and GC-MSCs increased about 32 and 7 folds compared to normal MSCs and GCN-MSCs, respectively (Fig. 1C). Furthermore, the m^6^A peak levels of CSF2 showed an approximate 3-fold increase in both P-MSCs and GC-MSCs compared to normal MSCs and GCN-MSCs, respectively (Fig. 1D). ELISA analyses of CSF2 protein levels in the supernatant indicated approximately 2.5-fold and 7.5-fold higher levels in P-MSCs and GC-MSCs than normal MSCs and GCN-MSCs, respectively (Fig. 1E). TCGA database analysis also revealed a higher level of CSF2 in gastric cancer specimens than normal gastric tissues (Fig. S1C). Immunohistochemical analysis of pathological sections from gastric cancer patients who underwent surgical treatment demonstrated predominant CSF2 expression at the base of gastric glands in mucosal tissues (Fig. 1F). Immunofluorescence results showed that MSC marker α-SMA was also predominantly localized at the base of gastric glands and co-localized with CSF2 in gastric cancer tissues (Fig. S1D). The specimens were categorized into two groups based on patient recurrence time. In patients who experienced relapse within 1 year after surgery, the positive rate of CSF2 in gastric cancer tissues was approximately 46%. In patients who did not experience relapse within 3 years after surgical treatment, the positive rate of CSF2 in gastric cancer tissues was approximately 9% (Fig. 1F). In summary, these findings indicate that the m^6^A modification and gene expression of CSF2 increase in tumor-conditioned MSCs and a high level of CSF2 in gastric cancer tissues is linked to poor prognosis.

### CSF2 induces the transition of MSCs into a tumor-promoting phenotype

**Fig. 2:**
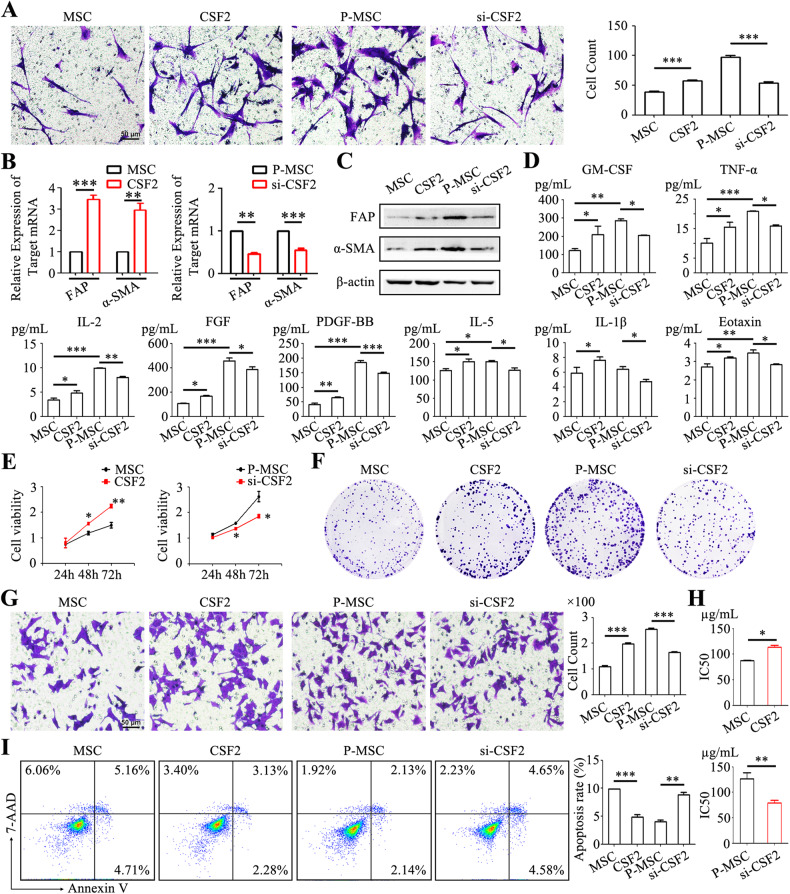
CSF2 regulates the pro-tumor phenotype and function of MSCs. **A** In vitro tropism of MSCs towards HGC-27 cells assessed via a transwell system. Original magnification: ×100. Scale bar = 50 μm. **B** qRT-PCR analysis of FAP and α-SMA expression in MSCs and P-MSCs. **C** Western blot analyses of FAP and α-SMA expression in MSCs and P-MSCs. **D** Luminex assay to determine the inflammatory cytokine profile in the supernatants from MSCs and P-MSCs. **E** Cell viability assay for HGC-27 cells treated with the supernatants from MSCs and P-MSCs. **F** Colony formation assays for HGC-27 cells treated with the supernatants from MSCs and P-MSCs. **G** Transwell migration assay for HGC-27 cells treated with the supernatants from MSCs and P-MSCs. Original magnification: ×100. Scale bar = 50 μm. **H** CCK-8 assay for IC50 of 5-FU in HGC-27 cells treated with the supernatants from MSCs and P-MSCs. **I** Flow cytometry analyses of the apoptotic rate of HGC-27 cells exposed to 5-FU. *P < 0.05; **P < 0.01; ***P < 0.001.

To elucidate the biological role of CSF2 in regulating MSC phenotype and function, we conducted CSF2 overexpression in normal MSCs and CSF2 knockdown in P-MSCs. In MSCs with CSF2 overexpression, a notable enhancement in tropism towards gastric cancer cells (57.33 ± 1.96) was observed, compared to control group (38.67 ± 1.86) (Fig. 2A). The mRNA and protein levels of FAP and α-SMA in MSCs were increased by CSF2 overexpression (Fig. 2B, C). Conversely, CSF2 knockdown suppressed the tropism of P-MSCs towards gastric cancer cells (53.83 ± 2.06 in CSF2 knockdown group and 97.17 ± 2.91 in control group) (Fig. 2A). Similarly, the expression of FAP and α-SMA was downregulated in CSF2 knockdown P-MSCs compared to control P-MSCs (Fig. 2B-C). CSF2 overexpression in MSCs resulted in elevated generation of GM-CSF, TNF-α, FGF, PDGF-BB, and IL-1β (Fig. 2D), whereas the secretion of these factors decreased when CSF2 was knocked down in P-MSCs (Fig. 2D).

The supernatants from MSCs with CSF2 overexpression and P-MSCs with CSF2 knockdown were collected and used to treat human gastric cancer cells. Compared to that from control MSCs, the supernatant from MSCs with CSF2 overexpression promoted gastric cancer cell viability (Fig. 2E), cell colony formation (Fig. 2F) and migration (Fig. 2G). The IC50 of 5-FU in MSCs with CSF2 overexpression group (113.95 ± 2.38) was approximately 1.5-fold of that in control MSC group (87.45 ± 1.17) (Fig. 2H). The rate of apoptotic cells after 5-FU pretreatment in MSCs with CSF2 overexpression group (4.82 ± 0.77%) was approximately half of that in control MSC group (9.86 ± 0.08 %) (Fig. 2I). Conversely, compared with P-MSC group, CSF2 knockdown reduced the effects of P-MSCs in promoting gastric cancer cell viability (Fig. 2E), cell colony formation (Fig. 2F), migration (254.83 ± 9.82 vs. 165.00 ± 7.78) (Fig. 2G) and reducing IC50 of 5-FU (126.74 ± 4.56 vs. 79.26 ± 3.01) (Fig. 2H) and apoptotic cell rate (4.04 ± 0.50% vs. 8.85 ± 0.69 %) (Fig. 2I).

The similar effect was also observed in gastric cancer cells treated with the supernatants from GCN-MSCs and GC-MSCs. CSF2 overexpression in GCN-MSCs resulted in enhanced cell tropism (Fig. S2A) and increased expression of FAP and α-SMA (Fig. S2B). Conversely, CSF2 knockdown in GC-MSCs had the opposite effect (Fig. S2A, S2B). The supernatant from GCN-MSCs with CSF2 overexpression promoted HGC-27 cell migration (Figure S2C) while the promotion of HGC-27 cell migration by GC-MSC supernatant was reduced by CSF2 knockdown (Fig. S2C). Collectively, these findings indicate that CSF2 plays a critical role in regulating the pro-tumor phenotype and function of MSCs in gastric cancer.

### IGF2BP2 enhances the m^6^A modification and stability of CSF2 mRNA in MCSs

**Fig. 3:**
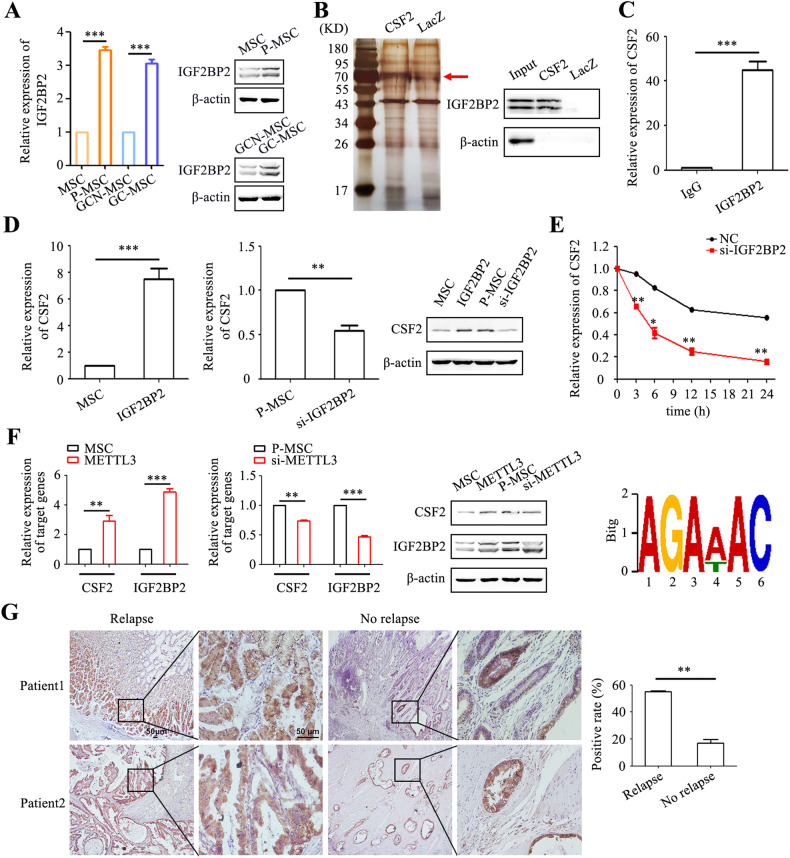
IGF2BP2 enhances the m^6^A modification and stability CSF2 mRNA in MSCs. **A** Quantification of IGF2BP2 expression in MSCs by qRT-PCR and western blot. **B** RNA pulldown assay for the interaction between CSF2 mRNA and IGF2BP2 protein. Proteins were identified through silver staining and western blot. **C** RIP assay for the interaction between CSF2 mRNA and IGF2BP2 protein. **D** Detection of CSF2 expression in MSCs and P-MSCs by qRT-PCR and western blot. **E** Assessment of CSF2 mRNA stability in P-MSCs with or without IGF2BP2 knockdown treated with actinomycin D at different time points. **F** Quantification of CSF2 and IGF2BP2 expression in MSCs with METTL3 modulation by qRT-PCR and western blot. **G** Immunohistochemical analyses of IGF2BP2 expression in gastric cancer tissues from patients with or without relapse. Magnification: ×40 and ×200. Scale bar = 50 μm. *P < 0.05; **P < 0.01; ***P < 0.001.

Our microarray results also showed a significant upregulation of IGF2BP2 protein, a key reader for m^6^A modification, in tumor-conditioned MSCs (Fig. S3A, B). We assessed the expression of IGF2BP2 in MSCs (Fig. 3A and S3E). Notably, IGF2BP2 expression was upregulated in gene and protein levels in P-MSCs and GC-MSCs compared to MSCs and GCN-MSCs, respectively (Fig. 3A). To investigate whether IGF2BP2 regulates CSF2, we designed a full-length CSF2 gene vector and conducted an RNA pulldown experiment. Silver staining revealed a protein band around 70 kDa in the CSF2 group (Fig. 3B), and subsequent western blot analysis confirmed the binding of IGF2BP2 to CSF2 mRNA (Fig. 3B). The results of RNA immunoprecipitation (RIP) showed approximately a 45-fold enrichment of CSF2 mRNA in the IGF2BP2 antibody group compared to the IgG control group (Fig. 3C), further supporting the interaction between IGF2BP2 and CSF2 mRNA. Furthermore, IGF2BP2 overexpression in MSCs led to increased expression of CSF2 at both gene and protein levels, while IGF2BP2 knockdown in P-MSCs resulted in decreased CSF2 expression (Fig. 3D). Notably, IGF2BP2 knockdown significantly reduced the half-life of CSF2 mRNA in P-MSCs, indicating that IGF2BP2 regulates the mRNA stability of CSF2 (Fig. 3E). Given that METTL3 is one of the most common m^6^A methyltransferases, we overexpressed METTL3 in MSCs and observed increased expression of both CSF2 and IGF2BP2 at gene and protein levels. Conversely, knockdown of METTL3 in P-MSCs inhibited the expression of CSF2 and IGF2BP2 (Fig. 3F). Our m^6^A-seq analysis unveiled that the m^6^A peak in CSF2 was primarily located at the position of 132075875-132076023 on the short arm of chromosome 5, which harbored the AGAWAC motif (Fig. 3F). TCGA data analysis showed an increased expression of IGF2BP2 in gastric cancer tissues compared to normal tissues (Fig. S3C). Furthermore, the expression of IGF2BP2 positively correlated with that of CSF2 in gastric cancer tissues with a correlation coefficient of 0.54 (Fig. S3D). The positive rate of IGF2BP2 was approximately 55% in gastric cancer patients who experienced relapse within 1 year after surgery, while it was approximately 17% in patients without recurrence within 3 years (Fig. 3G). Collectively, these findings suggest that IGF2BP2 enhances the mRNA stability of CSF2 and promotes its expression in MSCs through an m^6^A-dependent manner.

### IGF2BP2-mediated m^6^A modification of CSF2 is critical for MSC reprogramming

**Fig. 4:**
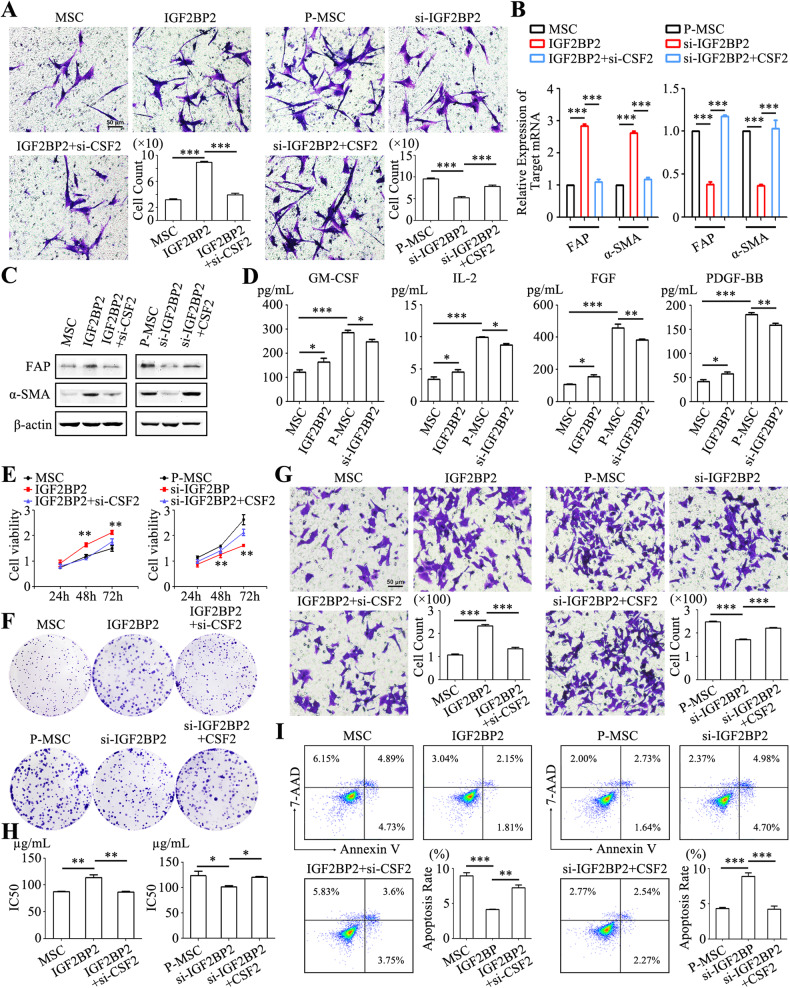
IGF2BP2 modulates CSF2 m^6^A modification to induce MSC reprogramming. **A** In vitro tropism of MSCs towards HGC-27 cells assessed via a transwell system. Original magnification: ×100. Scale bar = 50 μm. **B**, **C** qRT-PCR (**B**) and western blot (**C**) analyses of FAP and α-SMA expression in MSCs from different groups. **D** Luminex analyses of the inflammatory factor profile in the supernatants from MSCs. **E**, **F** The proliferation of HGC-27 cells following exposure to the supernatants from different MSCs was assessed by cell viability (**E**) and colony formation assays (**F**). **G** Transwell assay for the migration of HGC-27 cells upon treatment with the supernatants from different MSCs. Original magnification: ×100. Scale bar = 50 μm. **H** CCK-8 assay for IC50 of 5-FU in HGC-27 cells treated with the supernatants from different MSCs. **I** Flow cytometric analyses of the apoptotic rate of HGC-27 cells after exposure to 5-FU. *P < 0.05; **P < 0.01; ***P < 0.001.

We further examined the potential of IGF2BP2 in MSC reprogramming. IGF2BP2 overexpression in MSCs resulted in increased tropism towards gastric cancer cells (Fig. 4A). IGF2BP2 overexpression also led to an increased expression of FAP and α-SMA in MSCs (Fig. 4B, C). Notably, these effects were reversed upon simultaneous knockdown of CSF2 (Fig. 4A–C). In addition, IGF2BP2 knockdown in P-MSCs significantly suppressed their tropism towards gastric cancer cells (Fig. 4A) and decreased FAP and α-SMA expression in P-MSCs (Fig. 4B, C). Remarkably, the inhibition by IGF2BP2 knockdown could be rescued by co-transfection with CSF2 (Fig. 4A–C). IGF2BP2 overexpression in MSCs promoted the production of GM-CSF, FGF, and PDGF-BB, while IGF2BP2 knockdown led to the opposite effect on P-MSCs (Fig. 4D). Compared to control MSC group, the supernatant from IGF2BP2 overexpression MSCs group promoted the proliferation (Fig. 4E, F), migration (Fig. 4G), and drug resistance (Fig. 4H, I) of gastric cancer cells. However, the tumor-promoting effects were attenuated upon the ablation of CSF2 in IGF2BP2 overexpression MSCs (Fig. 4E–I). In contrast, knockdown of IGF2BP2 in P-MSCs resulted in a diminution of its promoting effect on gastric cancer cell proliferation (Fig. 4E, F), migration (Fig. 4G), and drug resistance (Fig. 4H, I). Nevertheless, the re-introduction of CSF2 further rescued these effects (Fig. 4E–I). Hence, these findings indicate that CSF2 acts as a downstream target of IGF2BP2 to promote the reprogramming of MSCs in gastric cancer.

### CSF2 induces Notch1 ubiquitination to inactivate Notch signaling in MSCs

**Fig. 5:**
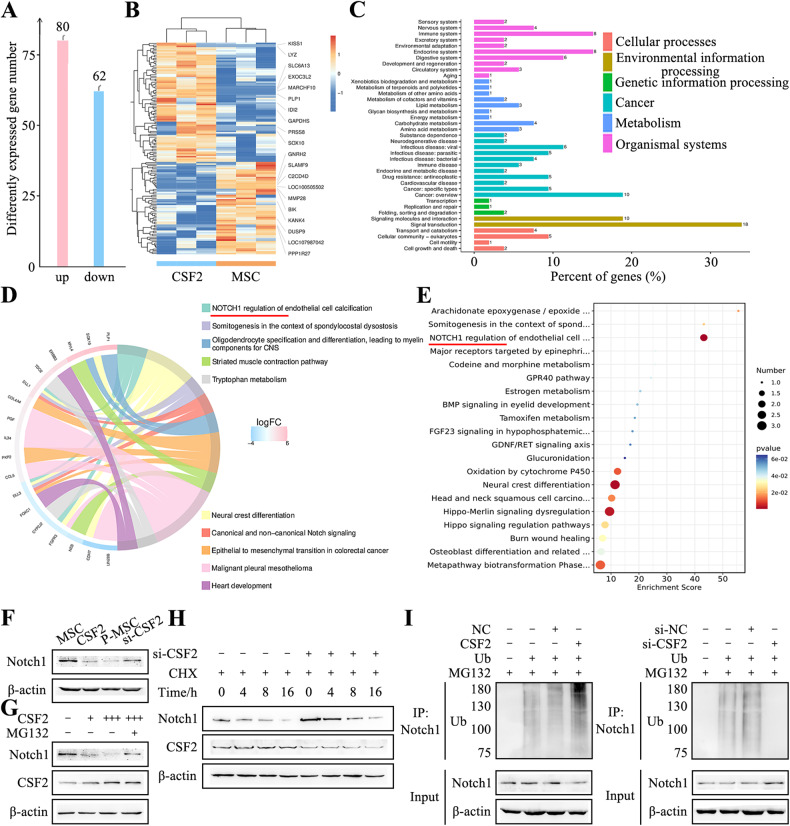
CSF2 induces Notch1 ubiquitination in MSCs. **A** Statistical representation of differentially expressed genes in control and CSF2-transfected MSCs. **B** Cluster diagram illustrating the grouping of differentially expressed genes. **C** KEGG analysis of the distribution of differentially expressed genes. **D** Chord chart analysis of the top10 genes based on the WikiPathways database. **E** Bubble plots depicting the enrichment of the top 20 downregulated genes according to WikiPathways. **F** Western blot analysis of Notch1 protein expression. **G** Western blot analysis of Notch1 protein levels in MSCs transfected with increasing doses of CSF2, with or without MG132 treatment. **H** CHX assay for the protein stability of Notch1 in control and CSF2 knockdown P-MSCs. **I** Assessment of Notch1 ubiquitination in ubiquitin (Ub)-transfected and MG132-treated MSCs (control and CSF2 overexpression) and P-MSCs (control and CSF2 knockdown). The immunoprecipitated ubiquitinated Notch1 proteins were detected using Notch1 and ubiquitin antibodies.

To elucidate the mechanism underlying CSF2-induced MSC reprogramming, we performed RNA sequencing to analyze the differentially expressed genes between control vector and CSF2-transfected MSCs. CSF2 overexpression led to the upregulation of 80 genes and downregulation of 62 genes in CSF2-transfected MSCs compared to control vector group (Fig. 5A). Notably, the top 30 differentially expressed genes, including KISS1, LYZ, and SLC6A13, were identified (Fig. 5B). KEGG analysis revealed that these top 30 genes were involved in multiple signaling pathways associated with cancer (Fig. 5C). Gene enrichment analysis and chord diagram visualization using the WikiPathways database showed that Notch1 signaling ranked first with the lowest *P*-value among the changed pathways (Fig. 5D). Furthermore, the genes of Notch signaling pathway showed downregulation in CSF2-transfected MSCs and the enrichment score for this pathway was significantly higher than other signaling pathways (Fig. 5E).

To validate the sequencing results, we examined the expression of receptor and ligand subtypes of the Notch pathway, as well as the target gene HES1 by qRT-PCR. CSF2 overexpression in MSCs resulted in decreased expression of Notch1 and HES1, while CSF2 knockdown in P-MSCs increased their expression. No significant differences were observed in the expression of other ligands or receptors of the Notch signaling pathway (Fig. S4). Western blot analysis of the intracellular fragment of Notch1 revealed that CSF2 downregulated whereas CSF2 knockdown upregulated Notch1 protein levels in MSCs (Fig. 5F). Pull-down experiments and mass spectrometry analysis did not reveal any physical interaction between CSF2 RNA and Notch1 protein. The inhibitory effect of CSF2 on Notch signaling was found to be independent of Notch1 nuclear translocation, suggesting that CSF2 may influence Notch1 function by regulating its protein level. Notch1 expression was significantly decreased by CSF2 overexpression, and this effect was inhibited by MG132, a specific proteasome inhibitor (Fig. 5G). Additionally, the results of cycloheximide (CHX) assay showed that the half-life of Notch1 protein in CSF2 knockdown P-MSCs was longer than that in control cells (>8 h vs. 4 h), suggesting that CSF2 regulates Notch1 protein at the post-translational level (Fig. 5H). Co-immunoprecipitation experiments followed by MG132 treatment were conducted to examine the levels of ubiquitinated Notch1 in MSCs co-transfected with CSF2 plasmid or siRNAs and Ubiquitin (Ub). The results showed that CSF2 overexpression significantly increased the levels of endogenous ubiquitinated Notch1, while CSF2 knockdown had the opposite effect (Fig. 5I). Therefore, these findings indicate that CSF2 induces Notch1 ubiquitination in MSCs.

### IGF2BP2/CSF2/Notch1 axis regulates MSC reprogramming in gastric cancer

**Fig. 6:**
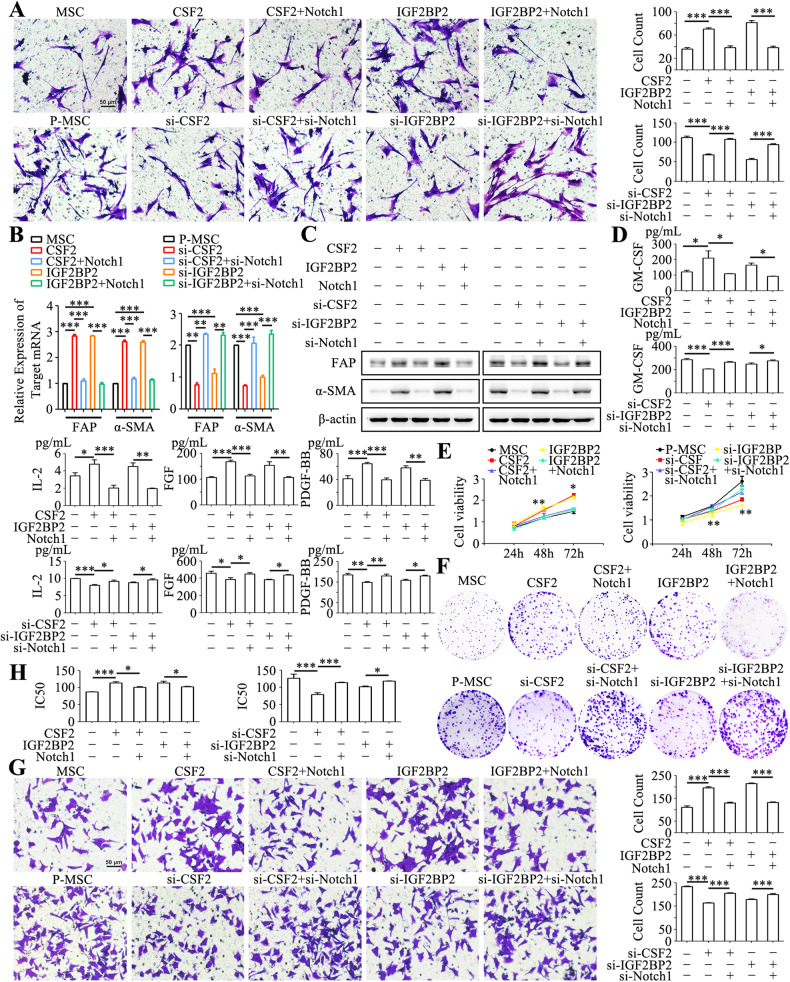
IGF2BP2/CSF2/Notch1 axis regulates MSC reprogramming. **A** In vitro tropism of MSCs towards HGC-27 cells assessed via a transwell system. Original magnification: ×100. Scale bar = 50 μm. **B**, **C** qRT-PCR (**B**) and western blot (**C**) were performed to analyze the expression of FAP and α-SMA in different MSCs. **D** Luminex assay was employed to examine the inflammatory factor profile in MSC supernatant from each group. **E**, **F** Cell viability assays (**E**) and colony formation assays (**F**) were performed to assess the proliferation of HGC-27 cells treated with the supernatants from different MSCs. **G** Transwell assays were performed to evaluate the migration of HGC-27 cells treated with MSC supernatant from each group. Original magnification: ×100. Scale bar = 50 μm. **H** CCK-8 assay for IC50 of 5-FU in HGC-27 cells. *P < 0.05; **P < 0.01; ***P < 0.001.

We next aimed to elucidate the involvement of IGF2BP2/CSF2/Notch1 axis in regulating the phenotype and function of MSCs in gastric cancer. Notch1 co-transfection partially reversed the effects of CSF2 on MSC phenotype and function, including the tropism towards gastric cancer cells (Fig. 6A), the enhanced FAP and α-SMA expression (Fig. 6B, C), and the secretion of GM-CSF, FGF, and PDGF-BB (Fig. 6D). Furthermore, the co-transfection of Notch1 inhibited the stimulatory effects of the supernatant from CSF2 overexpression MSCs on gastric cancer cell proliferation (Fig. 6E, F), migration (Fig. 6G) and drug resistance (Fig. 6H). Conversely, co-transfection of Notch1 inhibitors in P-MSCs with CSF2 knockdown showed rescued the inhibitory effect of CSF inhibitors, including augmented tropism towards gastric cancer cells (Fig. 6A), enhanced expression of FAP and α-SMA (Fig. 6B, C), elevated expression of inflammatory factors GM-CSF, FGF, and PDGF-BB (Fig. 6D). The inhibition of gastric cancer cell proliferation (Fig. 6E, F), migration (Fig. 6G), and drug resistance (Fig. 6H) by the supernatant from P-MSCs with CSF2 knockdown was also enhanced by Notch1 co-inhibition.

Similarly, co-transfection of Notch1 reversed the effects of IGF2BP2 on MSCs. Specifically, the introduction of Notch1 was found to mitigate the enhanced tropism towards HGC-27 cells (Fig. 6A), upregulated expression of FAP and α-SMA (Fig. 6B, C), and the production of GM-CSF, FGF, and PDGF-BB (Fig. 6D) by IGF2BP2 overexpression in MSCs. Furthermore, the introduction of Notch1 also curbed the augmentation of gastric cancer cell proliferation (Fig. 6E, F), migration (Fig. 6G), and drug resistance (Fig. 6H) by the supernatant from IGF2BP2 overexpression MSCs. Correspondingly, co-inhibition of Notch1 in P-MSCs reversed the effects of IGF2BP2 knockdown on MSCs reprogramming (Fig. 6A–D), as well as the effects of their supernatants on gastric cancer cell proliferation, migration, and drug resistance (Fig. 6E–H). Collectively, these results suggest an important role of IGF2BP2/CSF2/Notch1 axis in regulating MSCs reprogramming in gastric cancer.

### IGF2BP2/CSF2/Notch1 axis reprograms MSCs to promote gastric cancer progression in vivo

**Fig. 7:**
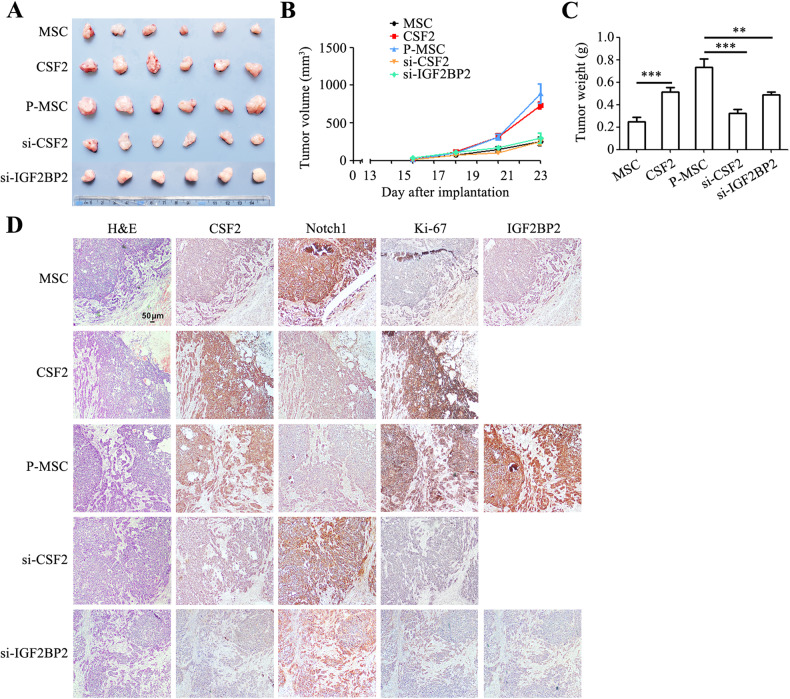
IGF2BP2/CSF2/Notch1 axis reprograms MSC to promote gastric cancer progression in vivo. **A** The images of tumors from mice that had received subcutaneous injection of HGC-27 cells and different MSCs (1:1 ratio). **B** Tumor growth curves of mice in different groups. **C** Tumor weights of mice in different groups. **P < 0.01; ***P < 0.001. **D** H&E, immunohistochemical analyses of Ki-67, CSF2, IGF2BP2, and Notch1 protein expression in tumor sections from different groups. Original magnification: ×40. Scale bar = 50 μm.

To further investigate the role of CSF2-mediated reprogramming of MSCs in promoting gastric cancer progression in vivo, we conducted subcutaneous xenograft mouse tumor models with HGC-27 cells together with MSCs from different sources (1:1 ratio). The tumors in CSF2 overexpression MSC group and P-MSC group exhibited significantly larger volumes than those in control MSC group, whereas the tumors in CSF2 knockdown and IGF2BP2 knockdown groups showed significantly smaller sizes than those in P-MSC group (Fig. 7A). The results of tumor growth curves showed that CSF2 overexpression MSC and P-MSC groups displayed faster tumor growth rates than control MSC groups, while CSF2 knockdown and IGF2BP2 knockdown in P-MSCs markedly reduced the tumor growth rate (Fig. 7B). At the end of experiment, the mean weight of tumors in CSF2 knockdown and IGF2BP2 knockdown groups were around 1/3 of those in P-MSC group (Fig. 7C). Immunohistochemistry results showed increased expression of Ki-67 and decreased expression of Notch1 in the CSF2 overexpression MSC group and P-MSC group. Notch1 expression was increased in the CSF2 knockdown and IGF2BP2 knockdown P-MSC groups compared to control P-MSC group. Moreover, the expression of IGF2BP2 was elevated in P-MSC group compared to MSC group (Fig. 7D).

To further validate these observations, we extended our analysis to cancer-derived MSCs and evaluated the impact of CSF2 and IGF2BP2 knockdown in GC-MSCs on tumor growth. The tumors from CSF2 knockdown and IGF2BP2 knockdown groups exhibited notably smaller sizes compared to tumors from GC-MSCs group (Fig. S5A). Moreover, the knockdown of CSF2 and IGF2BP2 in GC-MSCs significantly slowed the rate of tumor growth relative to GC-MSCs group (Fig. S5B). The weight of tumors in CSF2 knockdown and IGF2BP2 knockdown groups was approximately half of those in GC-MSCs group, further substantiating the roles of CSF2 and IGF2BP2 in maintaining the tumor-promoting roles of GC-MSCs (Fig. S5C). Immunohistochemical analyses of tumors in GC-MSCs group showed increased expression of CSF2 and IGF2BP2 and decreased Notch1 expression. Notably, CSF2 knockdown and IGF2BP2 knockdown in GC-MSCs resulted in elevated expression of Notch1 (Fig. S5D). Collectively, these findings indicate that IGF2BP2/CSF2/Notch1 axis reprograms MSCs to promote gastric cancer progression.

## Discussion

**Fig. 8:**
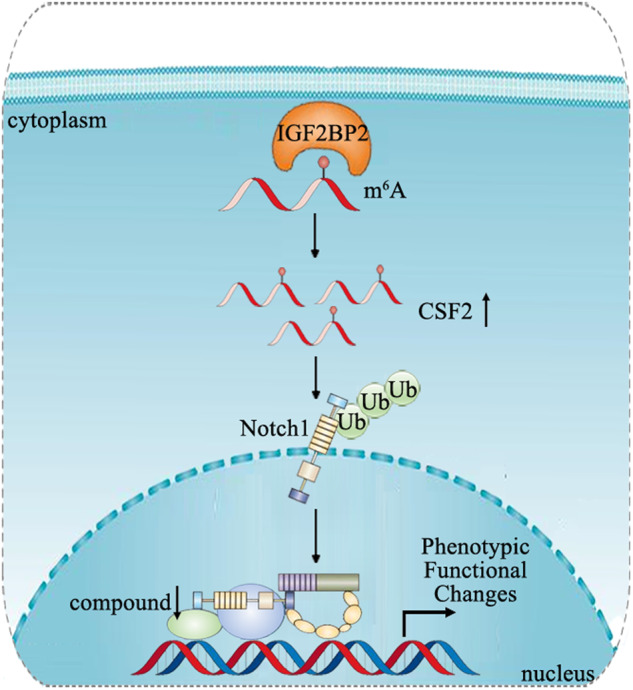
Proposed model for the role of IGF2BP2/CSF2/Notch1 axis in MSC reprogramming. The gastric cancer microenvironment induces increased expression of IGF2BP2, a crucial m^6^A reader protein, in MSCs. IGF2BP2 targets CSF2 in an m^6^A-dependent manner, enhancing the stability of CSF2 RNA and its expression. The upregulation of CSF2 leads to the ubiquitination of Notch1 and subsequent inhibition of Notch signaling. Consequently, MSCs undergo remodeling to acquire a tumor-promoting phenotype in gastric cancer.

MSCs play a crucial role in guiding the formation of tumor microenvironment. They undergo epigenetic reprogramming to acquire a tumor-promoting phenotype during tumor progression, which is known as carcinoma-associated MSCs. Emerging studies suggest that carcinoma-associated MSCs not only facilitate tumor growth and metastasis but also actively contribute to the evolution of tumor microenvironment, such as differentiation into other pro-tumorigenic matrix components, promotion of angiogenesis, and effective modulation of tumor immune exclusion [22]. In this study, we provided evidence showing that MSCs can be reprogrammed towards a pro-tumor phenotype by gastric cancer cells through an epigenetic mechanism, which involves increased IGF2BP2-mediated m^6^A modification and stability of CSF2 mRNA. The increase in CSF2 expression led to the ubiquitination of Notch1 and subsequent inactivation of Notch signaling (Fig. 8). These molecular events collectively reshaped the phenotype and function of MSCs, contributing to their pro-tumor properties.

Increasing studies highlight the reciprocal crosstalk between cancer cells and MSCs in tumor progression, adding new complexity and heterogeneity to tumor-stroma interactions. For instance, oxidized HMGB1 has been shown to recruit MSCs into tumors, thereby increasing colorectal cancer stemness and facilitating liver metastasis [23]. MSCs in hypoxic microenvironments promote liver cancer progression by activating YAP and the COX2/PGE_2_/EP4 axis [24]. Fan et al. demonstrated a novel model of ovarian cancer metastasis in which ovarian cancer cells reprogrammed the epigenome of MSCs, leading to the formation of carcinoma-associated MSCs and enabling co-metastasis of MSCs and cancer cells [25]. Lymph node metastasis-derived gastric cancer cells specifically educate MSCs through exosomal Wnt5a-mediated activation of YAP signaling [12]. Borella and colleagues discovered that AML cells remodeled resident MSCs by inducing transcriptome reprogramming, resulting in changes in the secretome and facilitating leukemia progression[26]. As the tumor grows, cancer cells secrete factors that can act in a paracrine manner to influence nearby tissue MSCs, inducing their transformation into carcinoma-associated MSCs, creating a more fertile “soil” for cancer cell propagation. We showed that tumor-conditioned MSCs produced increased amounts of inflammatory factors that have been previously demonstrated to promote cancer progression and metastasis via distinct mechanisms [27–32]. For example, GM-CSF was found to induce EMT through activation of MAPK/ERK or STAT3 signaling [27]. PDGF-BB bound to PDGFR-β and caused interaction of Notch1 with Furin, encouraging cell invasion and metastasis in pancreatic cancer [30]. IL-1β elicited ICAM1 expression by modulating intracellular ROS levels in head and neck squamous cell carcinoma, thereby fostering drug resistance [32]. Therefore, further elucidating the mechanisms underlying the transformation of normal MSCs into carcinoma-associated MSCs could facilitate the development of treatments to prevent or reverse these changes, thereby blocking the tumor-supportive properties of carcinoma-associated MSCs and potentially altering the entire tumor microenvironment.

In this study, we proposed a novel mechanism by which m^6^A modification regulates gene expression and downstream signaling in MSCs, leading to the reshaping of their phenotype and function. M^6^A modification is the most prevalent type of RNA modification observed in both mRNA and non-coding RNA. Recent studies have demonstrated the involvement of m^6^A in the differentiation of normal MSCs. For example, METTL3 and FTO have been implicated in the delicate balance between adipogenic and osteogenic differentiation of MSCs [33–35]. METTL14-mediated m^6^A modification of the ELMO1 3’UTR has been shown to enhance directional migration and osteogenic differentiation of MSCs, thereby improving the therapeutic efficacy in ankylosing spondylitis[36]. Additionally, MSCs have been found to enhance intestinal barrier function through METTL3/IGF2BP3-mediated m^6^A modification of pre-miR-34A during the repair of intestinal ischemia/reperfusion injury [37]. The function of m^6^A modification in the transformation of normal MSCs into carcinoma-associated MSCs remains limited. Pan et al. demonstrated that downregulation of METTL3 led to increased AKT protein levels in AML-MSCs, thereby enhancing MSC adipogenesis and contributing to chemoresistance in AML cells [38]. Herein, we identified IGF2BP2 as a regulator of the m^6^A modification of CSF2, which in turn mediated the transformation of carcinoma-associated MSCs, which provides novel insights into the biological roles of m^6^A modification in MSC reprogramming in cancer.

The upregulation of IGF2BP2 is associated with poor prognosis in various human cancers [39]. IGF2BP2 recognizes the m^6^A modifications and enhances the stability of target genes [39, 40]. Notably, IGF2BP2 has been found to target SIRT1 [41], ZEB1 [42], and HMGA1 [43], thereby promoting gastric cancer growth and metastasis. In this study, we showed that IGF2BP2 was highly expressed in carcinoma-associated MSCs and played a crucial role in reprogramming MSCs. We observed a direct physical interaction between IGF2BP2 and CSF2 mRNA and identified CSF2 as a novel target of IGF2BP2. Both the expression and the m^6^A modification level of CSF2 were found to be increased in reprogrammed MSCs. CSF2 was found to induce the regulation of fibrosis markers and pro-inflammatory factors in MSCs, consequently promoting tumor progression.

CSF2 has been demonstrated to target the receptor CD116, acting as an endogenous damage signal that facilitates multi-lineage differentiation and migration of BM-MSCs [44]. These findings suggest that CSF2 may function as a potential regulator of MSCs. Moreover, CSF2 has been implicated in the regulation of tumor microenvironment formation and promotion of tumor progression through immune-dependent and immune-independent mechanisms [45, 46]. CSF2 plays a crucial role in controlling the production, differentiation, and function of granulocytes and macrophages, and serves as a key mediator of the inflammatory response [47, 48]. For instance, CSF2 has been shown to attract and sustain the survival of microglia and macrophages in the glioblastoma microenvironment, promoting their pro-tumor polarization [49]. Additionally, myeloid-derived suppressor cells have been found to enhance the stemness of epithelial ovarian cancer cells through activation of the CSF2/p-STAT3 signaling pathway [50]. To the best of our knowledge, we provided here the first evidence of m^6^A modification of CSF2 and elucidated its role in MSC reprogramming.

Notch signaling pathway is composed of receptors, ligands, CSL-DNA binding proteins, and downstream effectors. The proteolytic cleavage of Notch ligands releases Notch protein fragments, which subsequently regulates gene expression. Notch signaling plays a crucial role in modulating the phenotype and function of MSCs. For instance, the activation of Notch signaling has been shown to enhance the migratory capacity of MSCs [51]. Additionally, the activation of Notch signaling by Jagged1 inhibits senescence and cell cycle arrest of MSCs [52]. Previous studies suggest that in the tumor microenvironment, Notch signaling serves as a molecular switch that regulates the plasticity of melanoma stem cells [53, 54]. In this study, we showed that CSF2 induced the ubiquitination and downregulation of Notch1 in MSCs and their subsequent pro-tumor phenotype and function, indicating a critical role of Notch1 in the reprogramming of MSCs in cancer.

In conclusion, our findings reveal a novel mechanism for MSCs reprogramming and tumor microenvironment remodeling. The identified IGF2BP2/CSF2/Notch1 axis provides additional evidence for the epigenetic regulation of MSCs within the tumor microenvironment and offers a new therapeutic strategy for gastric cancer.

## Materials and methods

### Cell culture

Human gastric cancer cell line HGC-27 was purchased from Cell Bank of the Chinese Academy of Sciences (Shanghai, China) and maintained in RPMI 1640 medium (Wisent, Canada) supplemented with 10% fetal bovine serum (FBS, ExCell, China). MSCs were isolated using the tissue adherent method [14] and characterized by FACS analyses and induced differentiation assays. MSCs pre-treated with conditioned medium from gastric cancer cells were termed as P-MSCs. MSCs obtained from gastric cancer tissues and adjacent tissues were named as GC-MSCs and GCN-MSCs, respectively. All cells were cultured at 37 °C in a humidified atmosphere of 5% CO_2_.

### Tissue samples

Umbilical cord tissues, gastric cancer tissues, and non-cancerous tissues were obtained from the Affiliated People’s Hospital of Jiangsu University. Informed consent was obtained from all patients who participated in the study, and the study protocol was approved by the Institutional Ethical Committee of Jiangsu University. All cancer patients included in the study had not undergone any radiotherapy or chemotherapy prior to surgical treatment. The pathological sections were categorized into two groups based on the time interval of recurrence: those with recurrence within 1 year and those without recurrence within 3 years (*n* = 3/group).

### Animal model

Animal experiments were conducted in compliance with the guidelines for animal care approved by the Jiangsu University Experimental Animal Management Committee. 48 male BALB/c nu/nu mice (Slake Experimental Animal Center in Shanghai) at the age of 4 weeks were randomly divided into 8 groups (*n* = 6/group). All groups received subcutaneous injections of HGC-27 (2 × 10^6^ cells in 200 μL PBS), Additionally, MSCs transfected with or without CSF2 plasmid, P-MSCs transfected with or without CSF2/IGF2BP2 inhibitor, and GC-MSCs transfected with or without CSF2/IGF2BP2 inhibitor were co-injected with HGC-27 cells in a 1:1 ratio. The mice were monitored every 2 days. Tumor volumes were calculated using the modified ellipsoidal formula: *V* = 1/2 (length × width^2^).

### RNA-seq and m^6^A-seq

Total RNA was extracted from distinct MSCs and subjected to RNA-seq analysis by Oebiotech (Shanghai, China) using the Illumina HiSeq platform. Quantitative analysis of all genes was performed using fragments per kilobase million (FPKM) values, and differentially expressed genes were identified based on criteria of |log_2_(FoldChange)| > 2 and a *P*-value < 0.05. For m^6^A-seq analysis, total poly(A)-selected RNA from MSCs and P-MSCs were used for m^6^A-specific RNA immunoprecipitation (RIP). Subsequently, RNA-seq was performed on the m^6^A-enriched RNA fraction.

### Plasmids, siRNA and gene transfection

The plasmids and small interfering RNA (siRNA) targeting CSF2, IGF2BP2, METTL3, and Notch1 (GenePharma, Shanghai, China) were transfected into cells using HiPerFect Transfection Reagent (Qiagen, Germany) according to the manufacturer’s instructions. The amounts of plasmids and siRNAs were 3 µg and 200 pmol, respectively. Sequences and modifications of the oligonucleotides are shown in Supplementary Table 1.

### CCK-8, plate cloning, and transwell assays

For CCK-8 assay, HGC-27 cells (3 × 10^3^ cells/well) were seeded in 96-well plates and treated with cell supernatant from different groups. After incubation for different times, each well was added with 10% CCK-8 solution. OD values were measured at a wavelength of 450 nm. The cell viability was calculated using the following formula: (measured value − blank value)/(control value − blank value) × 100%. For IC50 study, HGC-27 cells (3 × 10^3^ cells/well) were cultivated in 96-well plates and exposed to varying concentrations of 5-FU. The IC50 of 5-FU was calculated according to the resultant cell survival rate. For plate cloning assay, HGC-27 cells (1 × 10^3^ cells/well) were seeded in 6-well plates and treated with cell supernatant from different groups. Cells were fixed and stained with crystal violet at day 7.

For transwell assays, HGC-27 cells (5 × 10^4^ cells/well) suspended in serum-free medium were added to the top chamber of transwell plates. The bottom chamber of transwell plates (with 8-µm pore size; Corning, USA) contained cell supernatant from the respective experimental groups. After incubation at 37 °C for 8 h, the cells on the upper surface of the membrane were removed. The cells on the lower surface of the membrane were fixed and stained with crystal violet. The migration ability of the cells was determined by counting the cells in at least 6 fields for each experimental group.

For the tropism experiments, MSCs, GCN-MSCs, and GC-MSCs (5 × 10^4^ cells/well) with different treatments were suspended into the upper chamber of transwell plates. HGC-27 cells (1 × 10^5^ cells/well) were introduced into the lower chamber of the transwell plates. Following an incubation period of 24 h at 37 °C, the cells located on the upper surface of the membrane were removed and the cells on the lower surface of the membrane were fixed and subjected to crystal violet staining.

### Cell apoptosis assay

Early and late cell apoptosis was assessed using an Annexin V-PE/7-AAD apoptosis detection kit (BD Pharmingen, USA). The cells were treated with 5-FU (30 μg/mL) for 48 h, harvested, and analyzed using a BD FACS Calibur instrument. The number of cells in each category was quantified.

### Total RNA extraction and quantitative real time PCR analyses

Total RNA was extracted using Trizol reagent (Thermo, USA). Quantitative analysis of gene expression was performed using the HiScript 1st Strand cDNA Synthesis Kit and SYBR-Green I Real-Time PCR kit (Vazyme, China) on an ABI 7500 real-time PCR Detection System (Thermo). The expression levels were normalized to β-actin as a reference gene. The primer sequences used for mRNA analysis are provided in Supplementary Table 1.

### Western blot

Cell lysis was performed using RIPA buffer (Merck Millipore, USA) supplemented with protease inhibitors (Merck Millipore) to extract proteins. Equal amounts of protein samples were separated by SDS-polyacrylamide gel electrophoresis (SDS-PAGE) on a 12% polyacrylamide gel. Subsequently, the proteins were electrophoretically transferred onto 0.22 μm PVDF membranes (Merck Millipore), followed by blocking with 5% non-fat milk. Primary antibodies against FAP (#66562), α-SMA (#19245), Notch1 (#3608), CSF2 (#56712), ubiquitin (#20326) (Cell Signaling Technology, USA), and IGF2BP2 (11601-1-AP, Proteintech, Wuhan, China) were applied to the membranes for incubation. β-actin (CW0096M, Cwbio, Beijing, China) was used as the loading control. After incubation with an HRP-linked secondary antibody, the protein bands were visualized using chemiluminescence (Santa Clara, USA).

### RNA pulldown assay

Control and CSF2 RNA probes were synthesized by Biosense (Guangzhou, China). RNA pulldown was carried out in accordance with the manufacturer’s instructions (Biosense). Briefly, cells were lysed and exposed to streptavidin magnetic beads conjugated with RNA probes specific to the full-length CSF2. The CSF2-binding proteins were subsequently identified through mass spectrometry analysis and confirmed by western blot.

### LC–MS/MS

The protein samples underwent enzymatic digestion, desalting, and other necessary steps before liquid chromatography-mass spectrometry (LC-MS/MS) analysis. This analysis generated a raw file containing the original mass spectrometry data. The raw file was subsequently processed using Byonic software, which conducted a search against the UniProt-Homo Sapiens database to identify the proteins present in the samples.

### RNA immunoprecipitation assay

RNA immunoprecipitation (RIP) assay was conducted using the Magna RIP RNA-Binding Protein Immunoprecipitation Kit (Millipore, Bedford, MA). Cells were lysed in a lysis buffer supplemented with a protease inhibitor cocktail and RNase inhibitor. Magnetic beads were pre-incubated with anti-IGF2BP2, anti-rabbit IgG, or anti-m^6^A antibodies for 1 h at room temperature. Cell lysates were then subjected to immunoprecipitation with the beads overnight at 4 °C. RNA was subsequently purified from the immunoprecipitated complexes and subjected to qRT-PCR analysis.

### In vitro ubiquitin assays

In vitro ubiquitination system was prepared by combining Notch1 (5 μM), E1 (0.1 μM), E2 (2.5 μM), UB (3 μM), and 5 μL of a 10×ATP regeneration solution, resulting in a final volume of 50 μL. The system was incubated at 30 °C for 4 h. Subsequently, the ubiquitinated form of Notch1 was isolated using the same pulldown protocol as described previously. The presence of ubiquitinated Notch1 was then determined by western blot.

### Immunohistochemistry and immunofluorescence

The slides were subjected to antigen retrieval by boiling in citrate buffer (10 mM, pH 6.0). Subsequently, the slides were blocked with 5% bovine serum albumin (BSA; Boster Bioengineering, Wuhan, China). Primary antibodies against CSF2, IGF2BP2, Notch1 and Ki-67 (all at a 1:100 dilution ratio) were incubated with the slides at 4 °C overnight. Following this, the slides were incubated with a secondary antibody at 37 °C for 30 min. DAB (3,3’-diaminobenzidine) was used for visualization, and hematoxylin was used for counterstaining.

For immunofluorescence, the slides underwent sealing with goat serum after antigen retrieval treatment (Boster Bioengineering, Wuhan, China). Thereafter, the slides were incubated with anti-mouse α-SMA and anti-rabbit CSF2 antibodies (at a dilution ratio of 1:100) at 4 °C overnight. The slides were then incubated with FITC-conjugated anti-mouse and Cy3-conjugated anti-rabbit secondary antibodies at 37 °C for 1 h. The nuclei were visualized using Hoechst 33342 staining.

### Luminex assay and ELISA

The supernatants from MSCs in different groups were collected. The Bio-Plex Pro Human Cytokine Grp I Panel 27-plex kit (Catalog #M500KCAF0Y, Bio-Rad, USA) was used to detect the levels of GM-CSF, G-CSF, IL-1β, IL-1α, IL-2, IL-4, IL-5, IL-6, IL-7, IL-8, IL-9, IL-10, IL-12, IL-13, IL-15, IL-17A, MCP-1, TNF-α, VEGF, IFN-γ, IP-10, MIP-1α, MIP-1β, PDGF-BB, and CCL5 in different MSCs. All procedures were conducted following the manufacturer’s instructions. The signal was detected and analyzed using the Bio-Plex MAGPIX System (Bio-Rad, USA). For ELISA assay, all procedures were conducted following the manufacturer’s instructions (ExCell Bio, China).

### RNA stability assay

P-MSCs were transfected with si-IGF2BP2 and then treated with 10 µg/mL of actinomycin D (Sigma, Merck, Germany) in complete medium. Total RNA was extracted at 0, 3, 6, 12, and 24 h after treatment for subsequent qRT-PCR analysis.

### Statistical analysis

All results are the mean of three independent experiments, and data are presented as means ± SD. Statistical analysis was conducted using SPSS22 software. All continuous variables were tested for normal distribution. For comparisons among multiple groups, analysis of variance (ANOVA) followed by the least significant difference (LSD) post hoc test was applied. For comparisons between two groups, the Student’s *t*-test was used. The correlation between groups was assessed using Pearson’s correlation coefficient. *P* < 0.05 was considered statistically significant.

## Data and material availability

All data associated with this study are present in the paper or the Supplementary Materials.

### Supplementary information

Supplementary Figure legends

Figure S1

Figure S2

Figure S3

Figure S4

Figure S5

Supplementary Table 1

Original Data File
