# Supplementary material for: IGF2BP2-meidated m6A modification of CSF2 reprograms MSC to promote gastric cancer progression
Source: Cell Death Dis. 2023 Oct 21;14(10):693. doi: 10.1038/s41419-023-06163-7 (PMC10590395; doi:10.1038/s41419-023-06163-7)
Supplement: Supplementary file 1 — Supplementary Figure legends [file 41419_2023_6163_MOESM1_ESM.docx]

**Supplementary Figure legends**

**Supplementary Figure 1**

(A) Flow cytometric analyses of CD29, CD44, CD45, and CD90 expression on MSCs. (B) The induced differentiation potential of MSCs was determined by osteogenic and adipogenic differentiation experiments. (C) TCGA data analyses of the expression of CSF2 in gastric cancer tissues (n=408, group T) and normal gastric tissues (n=211, group N). (D) Immunofluorescence assay for the co-localization of α-SMA and CSF2 in gastric cancer tissues. Original magnification: × 40 and × 200. Scale bar = 50μm. **P*<0.05.

**Supplementary Figure 2**

(A) Transwell assays for the effect of CSF2 on the tropism of GCN-MSCs (control and CSF2 overexpression) and GC-MSCs (control and CSF2 knockdown) towards gastric cancer cells. Original magnification: × 100. Scale bar = 50μm. (B) qRT-PCR analyses of CSF2 on the expression levels of FAP, α-SMA, N-cadherin, and E-cadherin in MSCs. (C) Scratch assays were performed to assess the migratory ability of HGC-27 cells exposed to the supernatants from different MSCs. **P*<0.05; ***P*<0.01.

**Supplementary Figure 3**

(A) Cluster analysis was conducted to elucidate variations in the expression of RNA m^6^A-modifing proteins. (B) Significance in the expression levels of m^6^A-modifing proteins was determined through a volcanic map analysis. (C) TCGA data analyses of the expression of IGF2BP2 in gastric cancer tissues (n=408) and normal gastric tissues (n=211). (D) The correlation between the expression of CSF2 and IGF2BP2 was examined. (E) qRT-PCR analyses of the expression of IGF2BP1 and IGF2BP3 in different MSCs.

**Supplementary Figure 4**

Quantitative real-time PCR (qRT-PCR) was performed to analyze the expression levels of Notch receptor subtypes (Notch1/2/3/4), ligand subtypes (Jagged1/2, DLL1/3/4), and the target gene HES1 in different MSCs.

**Supplementary Figure 5**

(A) The images of tumors from mice that had received subcutaneous injection of HGC-27 cells and different GC-MSCs (1:1 ratio). (B) Tumor growth curves of mice in different groups. (C) Tumor weights of mice in different groups. ****P*<0.001. (D) H&E, immunohistochemical analyses of CSF2, IGF2BP2, and Notch1 protein expression in tumor sections from different groups. Original magnification: × 40. Scale bar = 50μm.
