# Supplementary figures and images for: IGF2BP2-meidated m6A modification of CSF2 reprograms MSC to promote gastric cancer progression
Source: Cell Death Dis. 2023 Oct 21;14(10):693. doi: 10.1038/s41419-023-06163-7 (PMC10590395; doi:10.1038/s41419-023-06163-7)

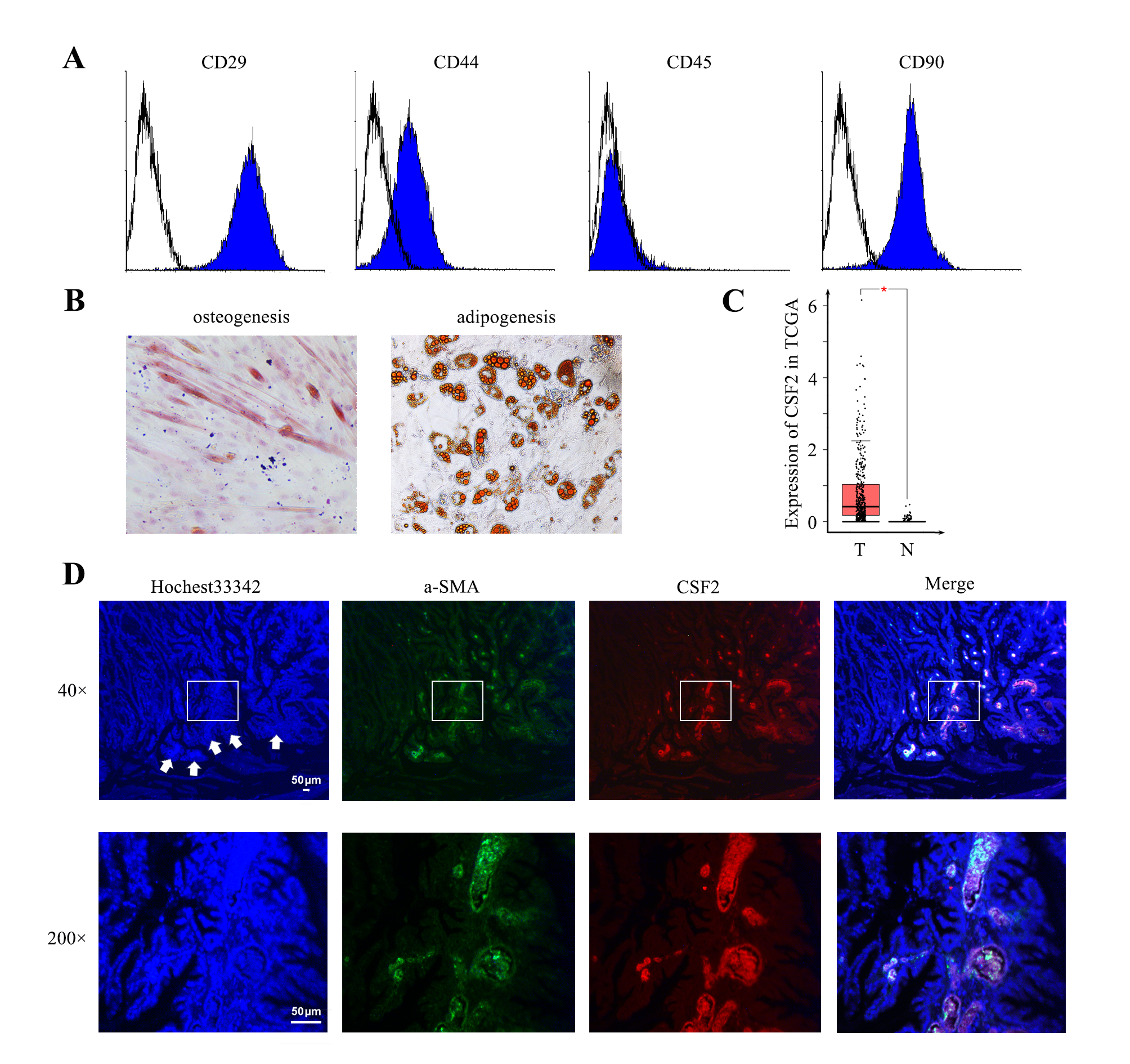

Supplement: Supplementary file 2 — Figure S1 [file 41419_2023_6163_MOESM2_ESM.tif]

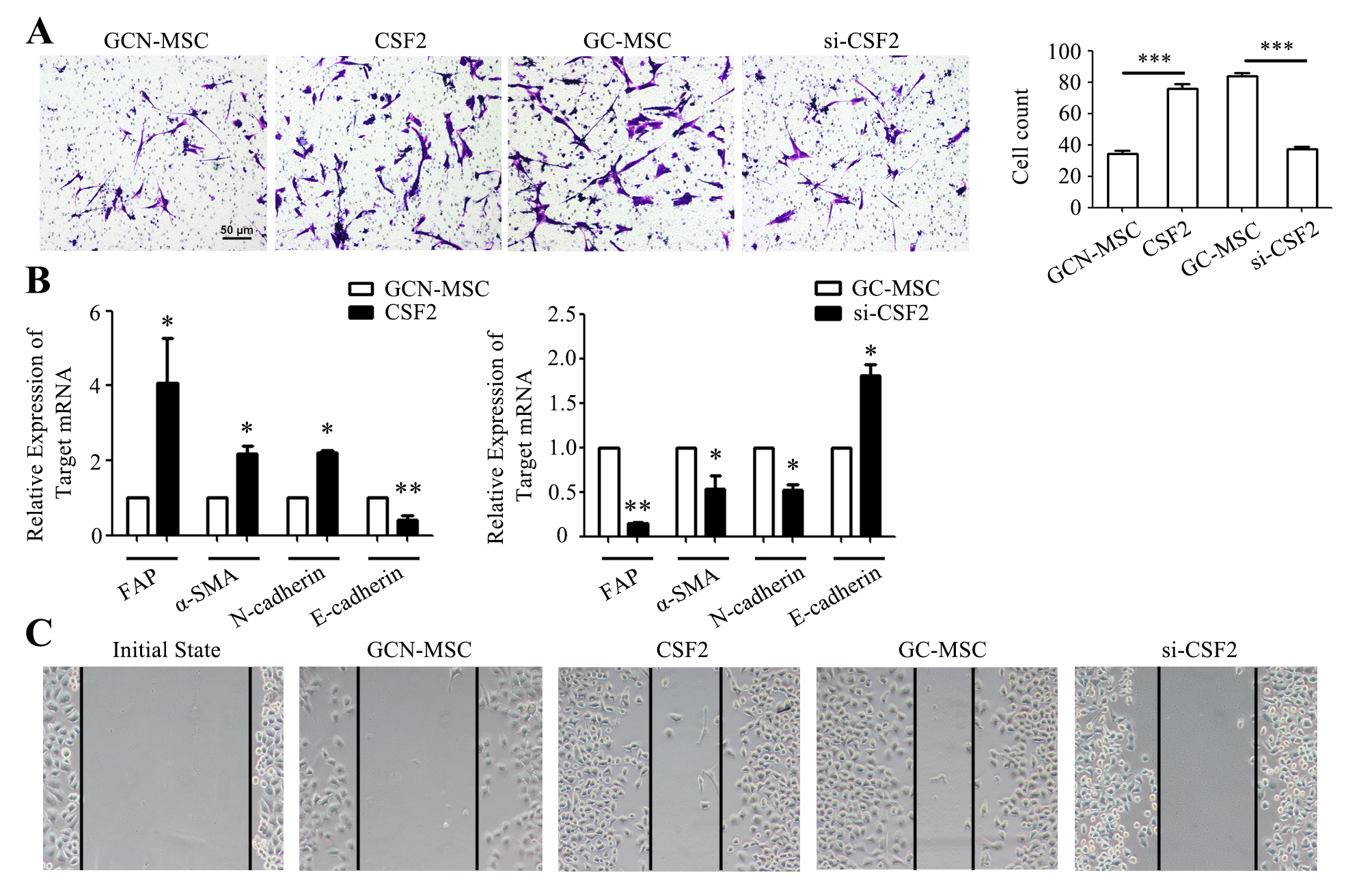

Supplement: Supplementary file 3 — Figure S2 [file 41419_2023_6163_MOESM3_ESM.tif]

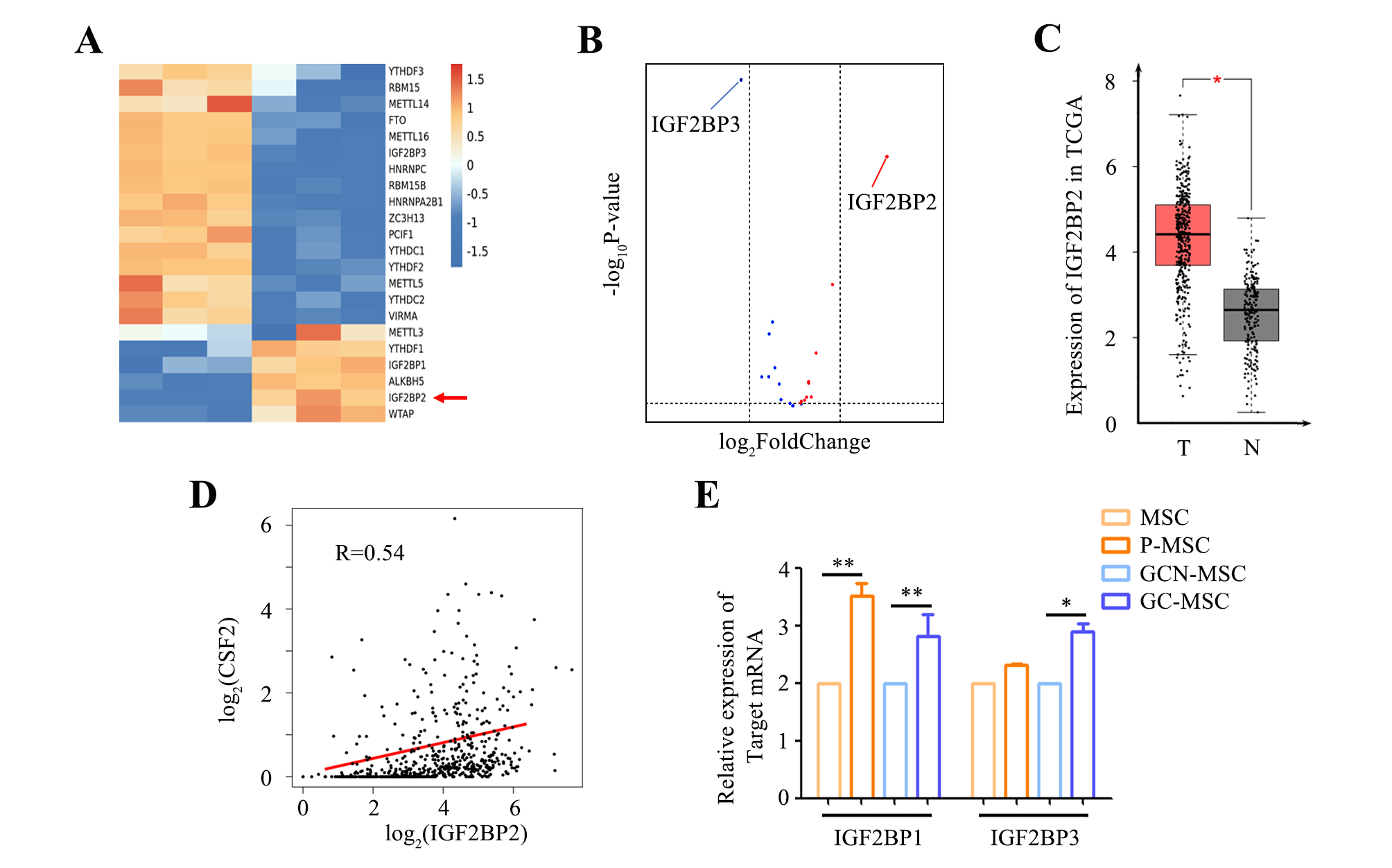

Supplement: Supplementary file 4 — Figure S3 [file 41419_2023_6163_MOESM4_ESM.tif]

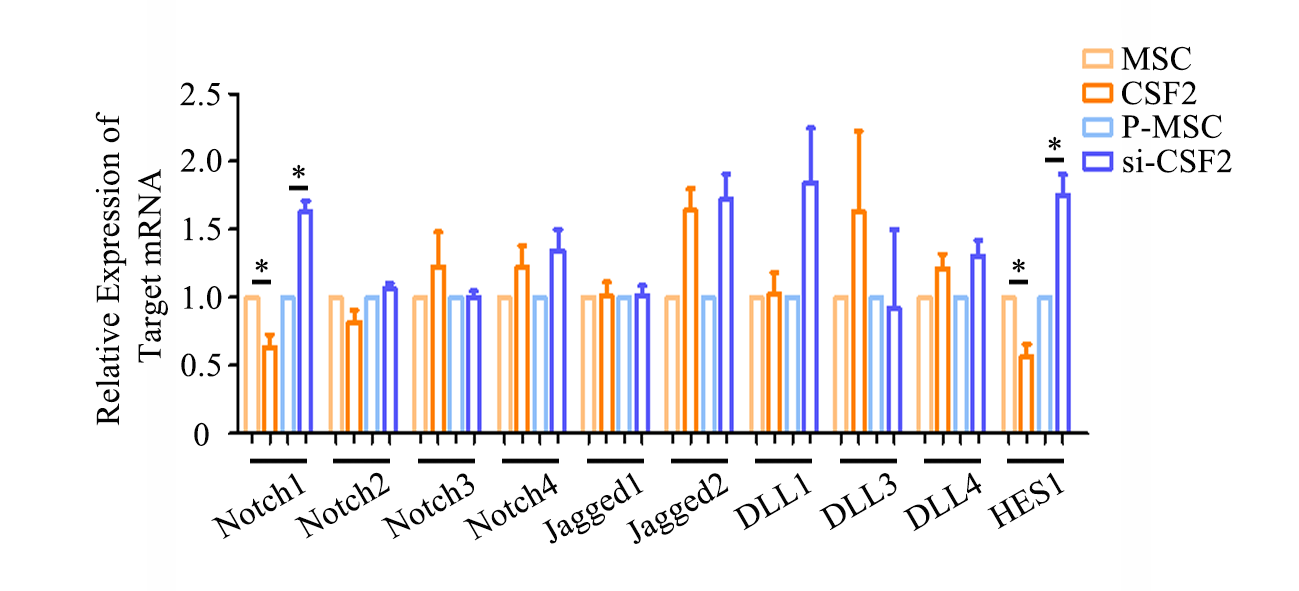

Supplement: Supplementary file 5 — Figure S4 [file 41419_2023_6163_MOESM5_ESM.tif]

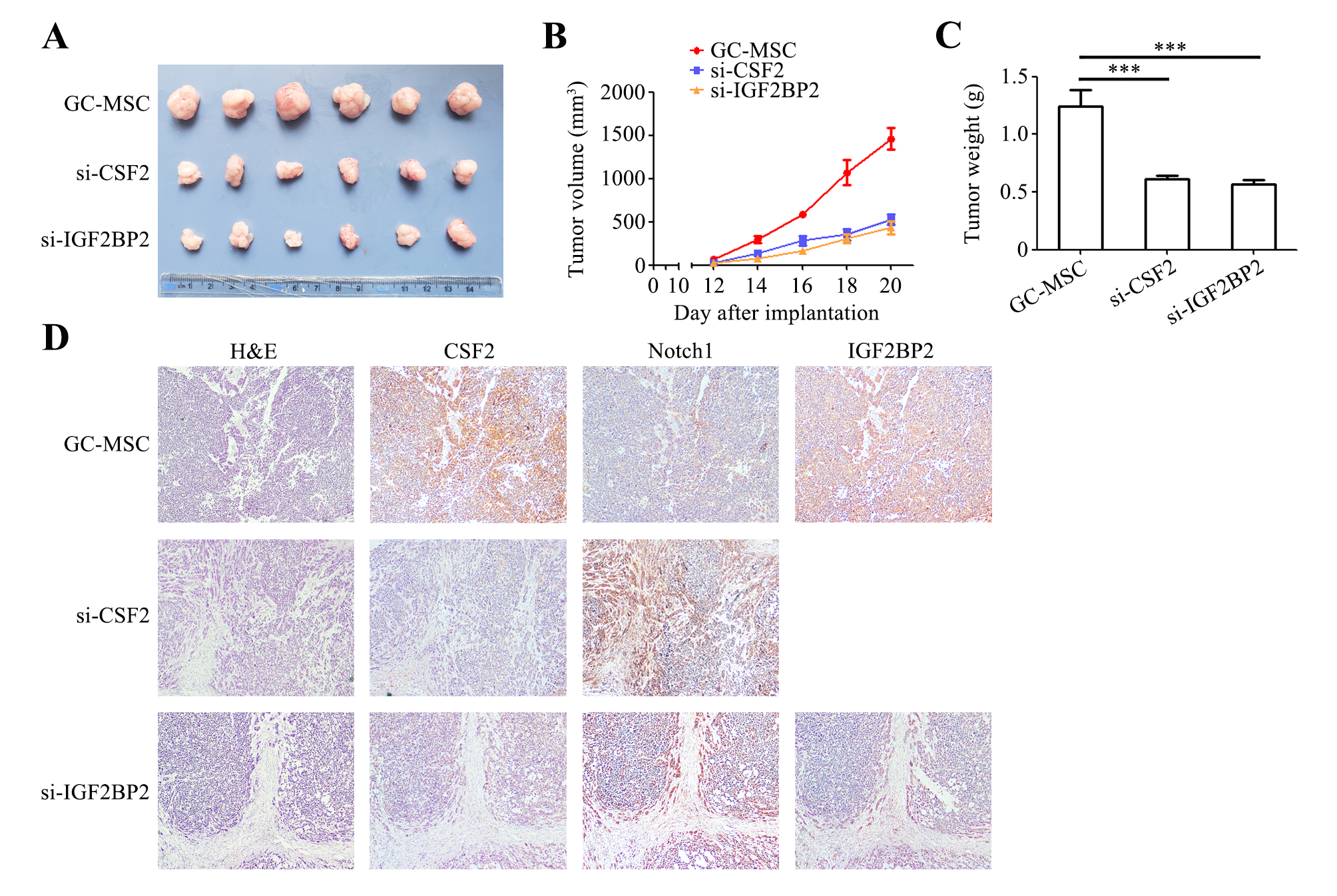

Supplement: Supplementary file 6 — Figure S5 [file 41419_2023_6163_MOESM6_ESM.tif]

Figure 2


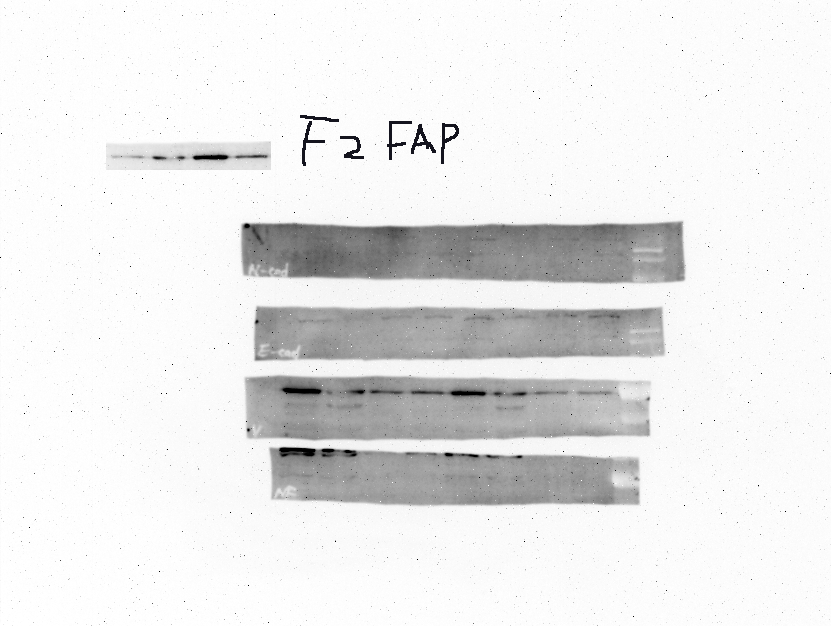

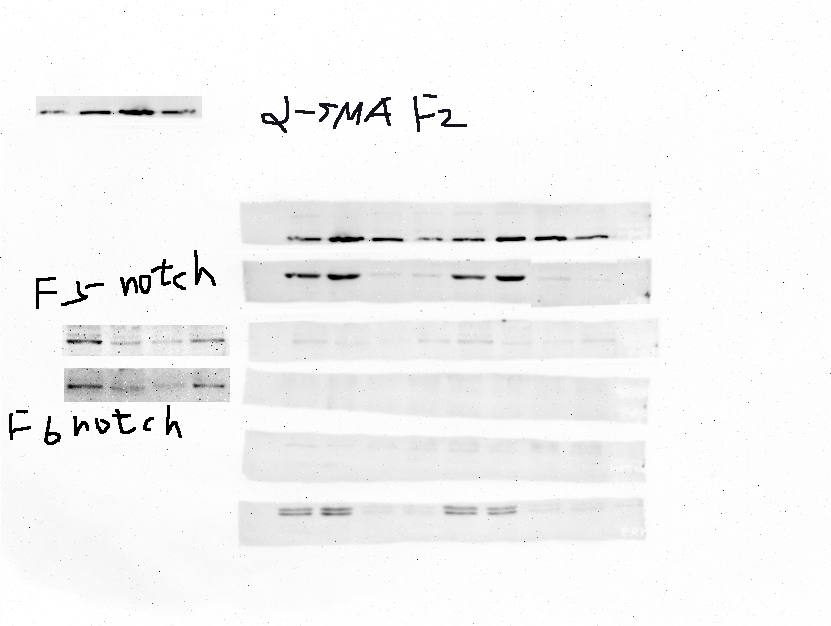

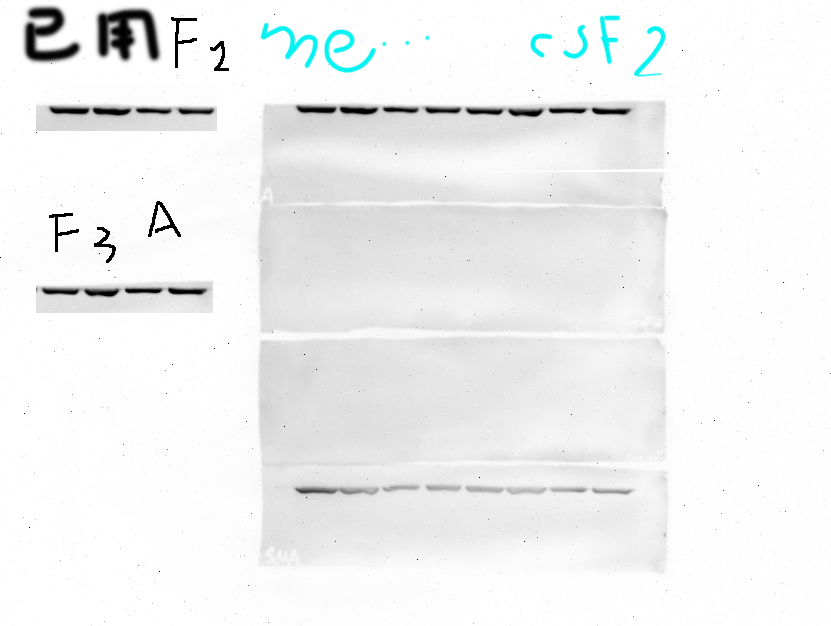


Figure 3




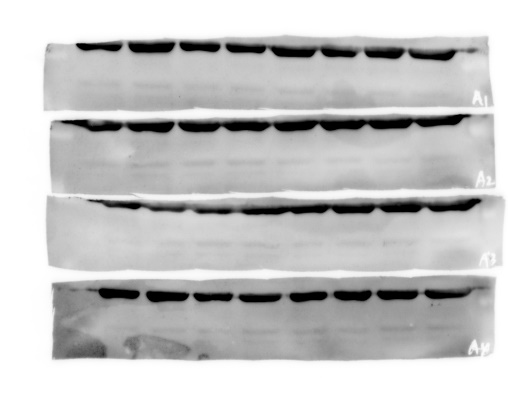

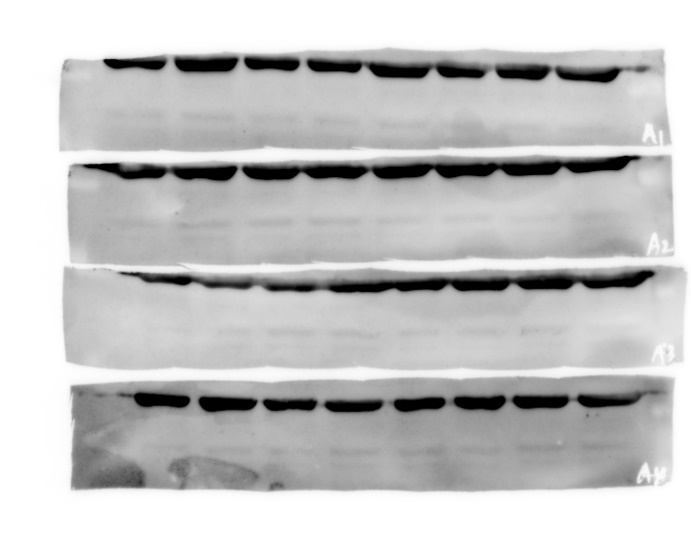



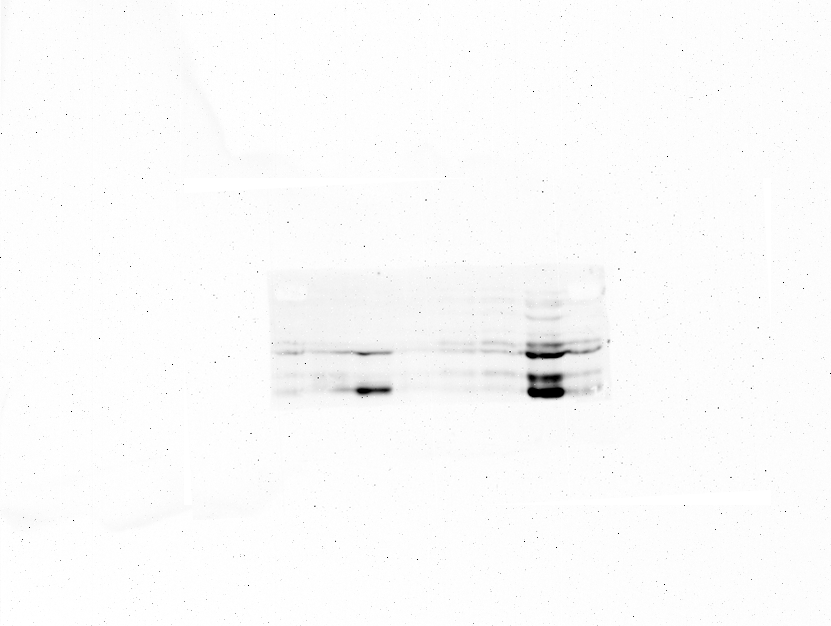

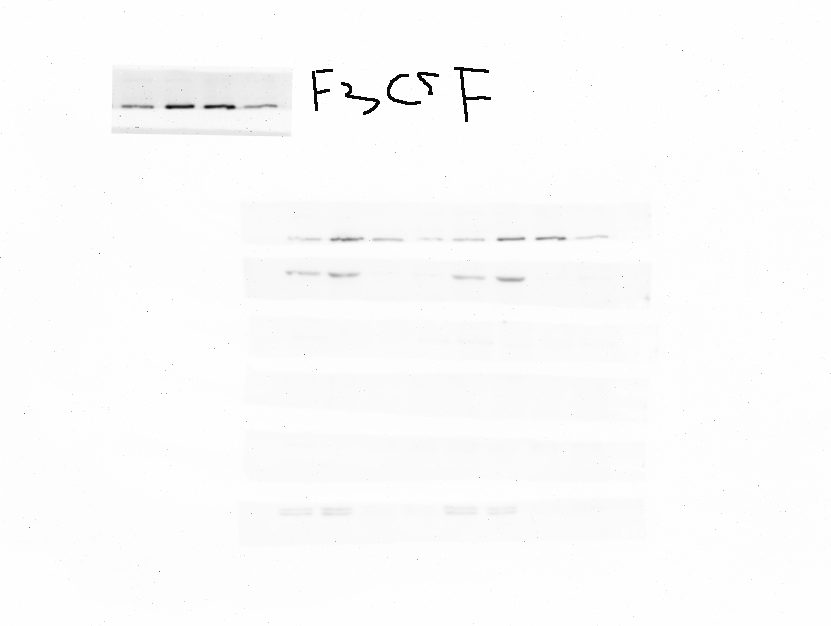

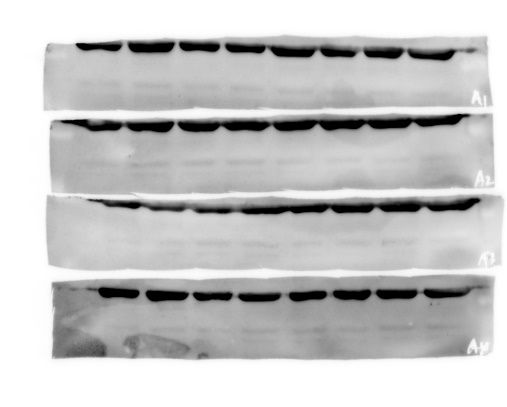





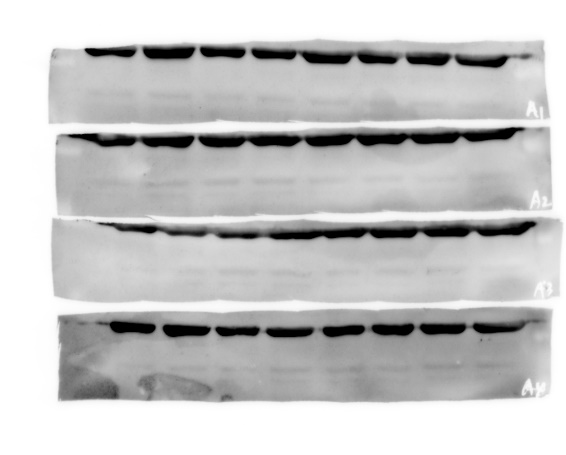


Figure 4


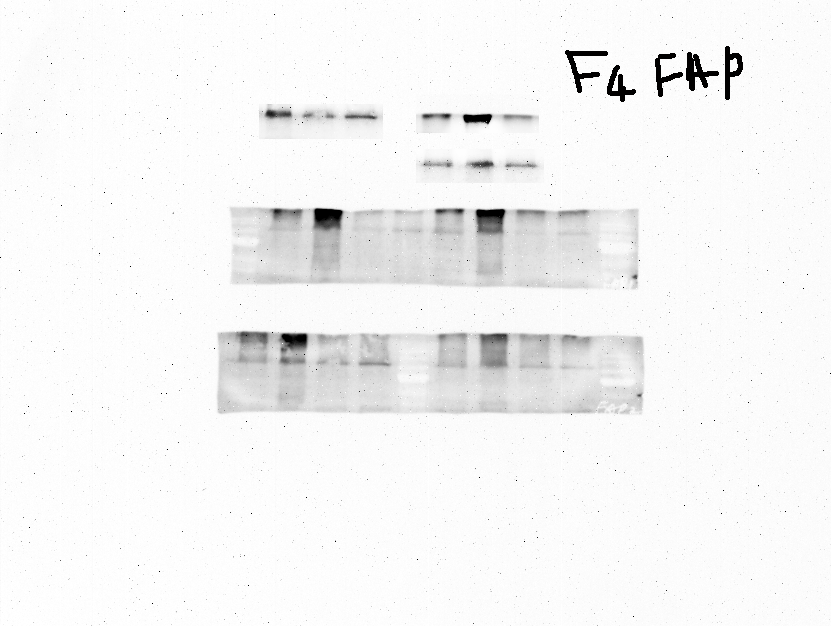

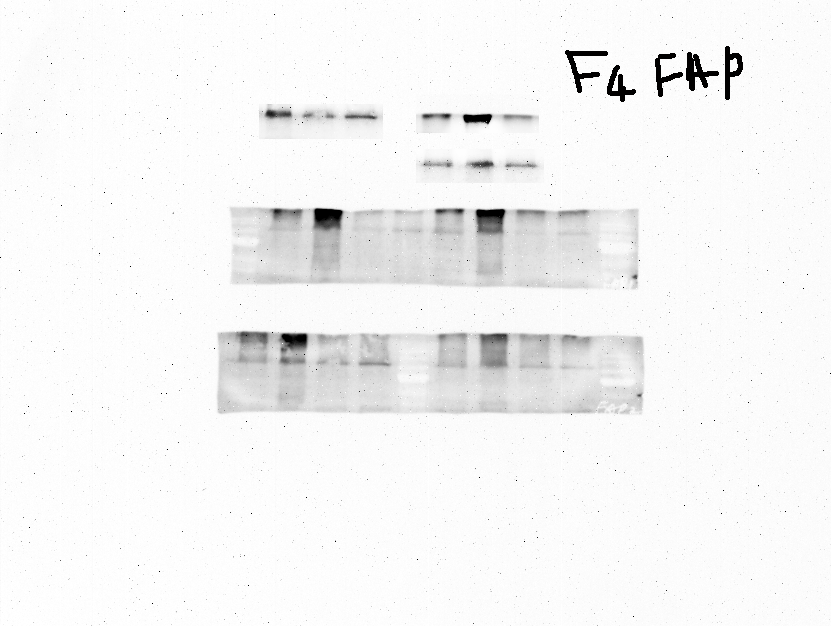

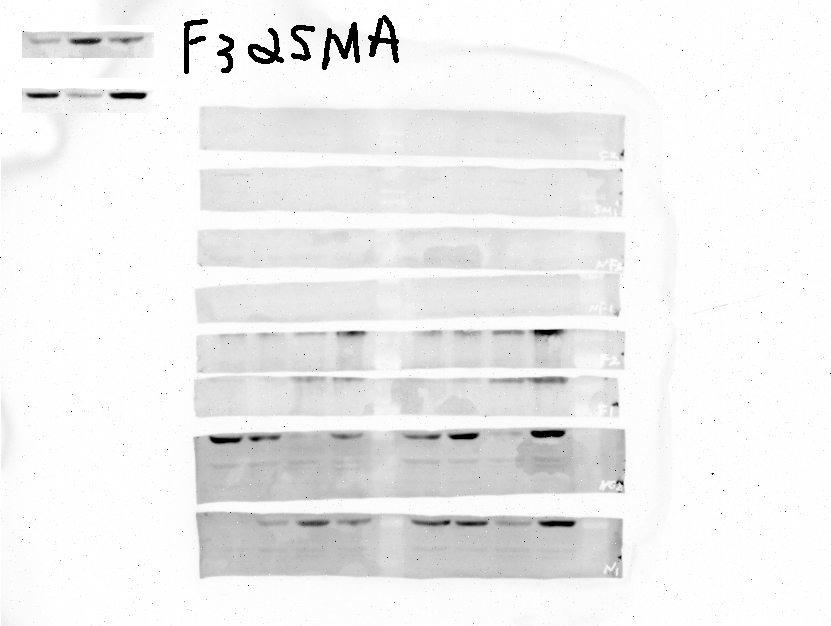

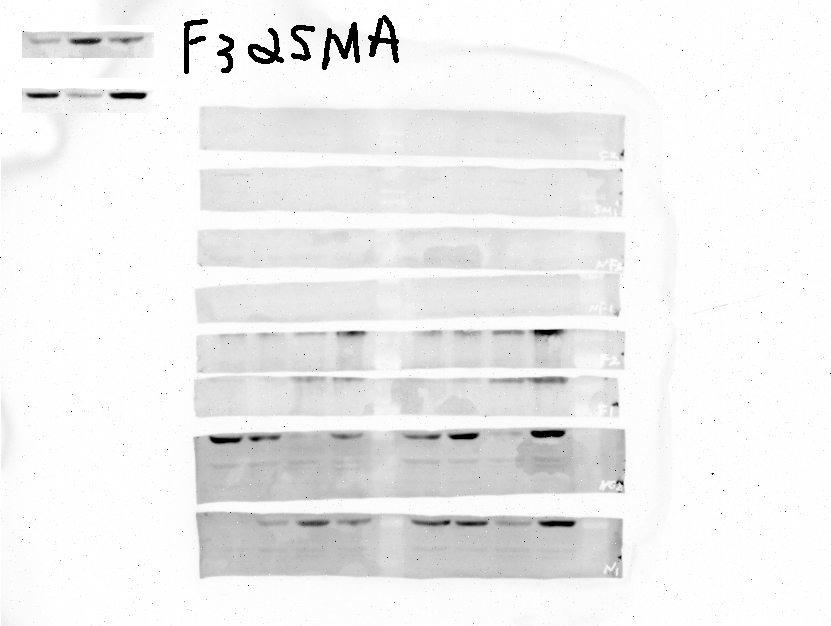

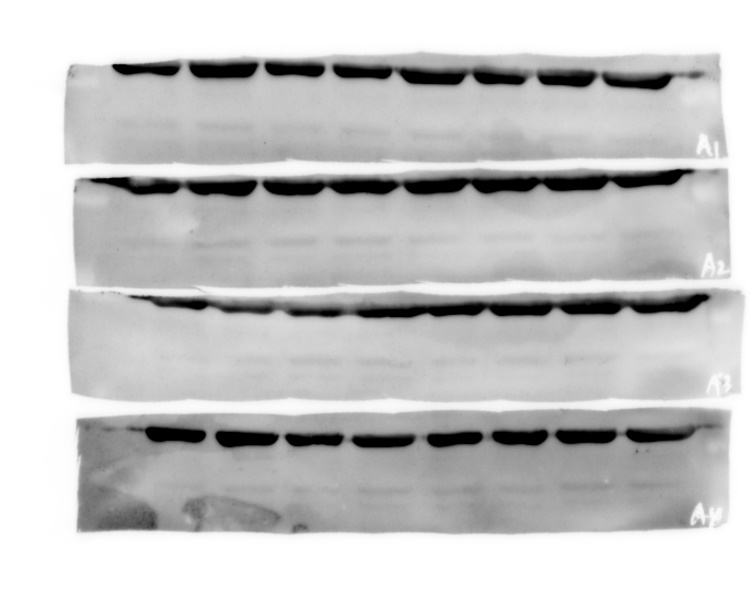

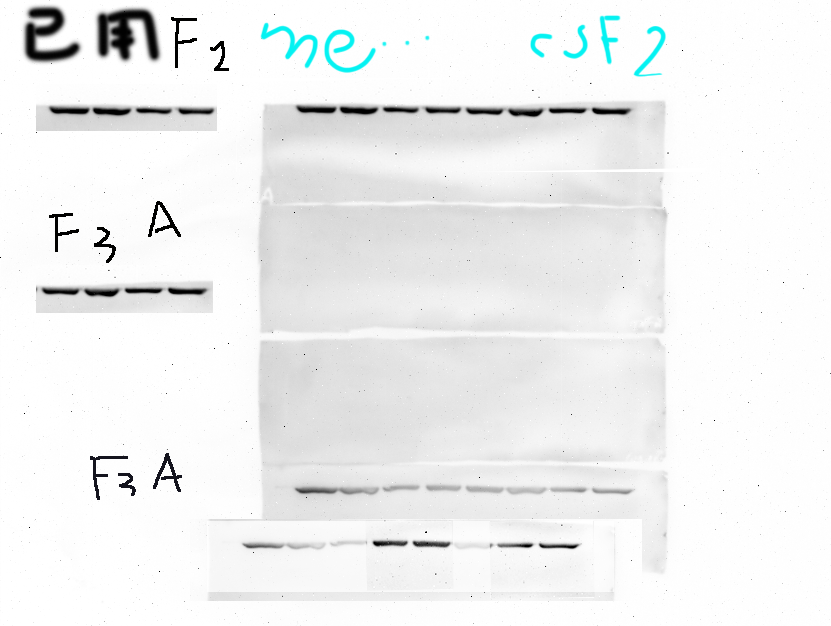


Figure 5


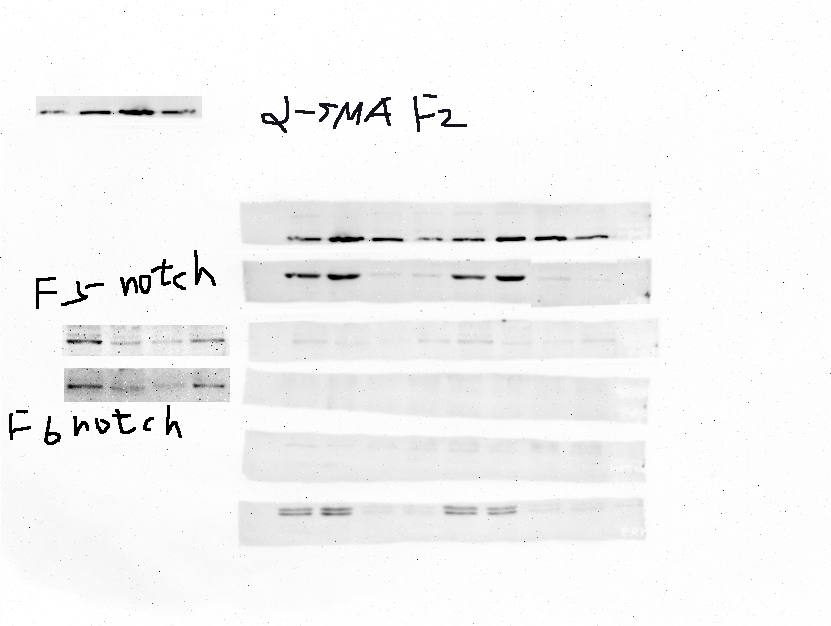



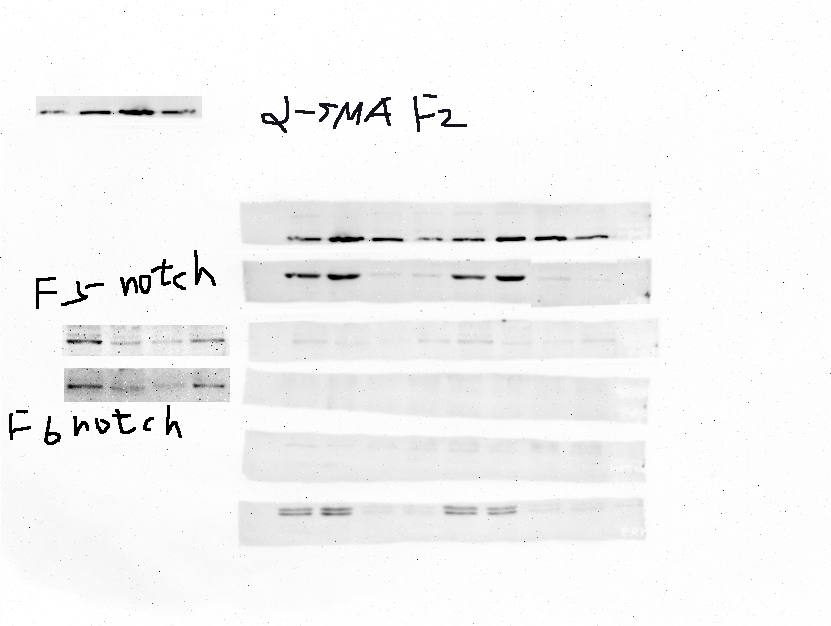













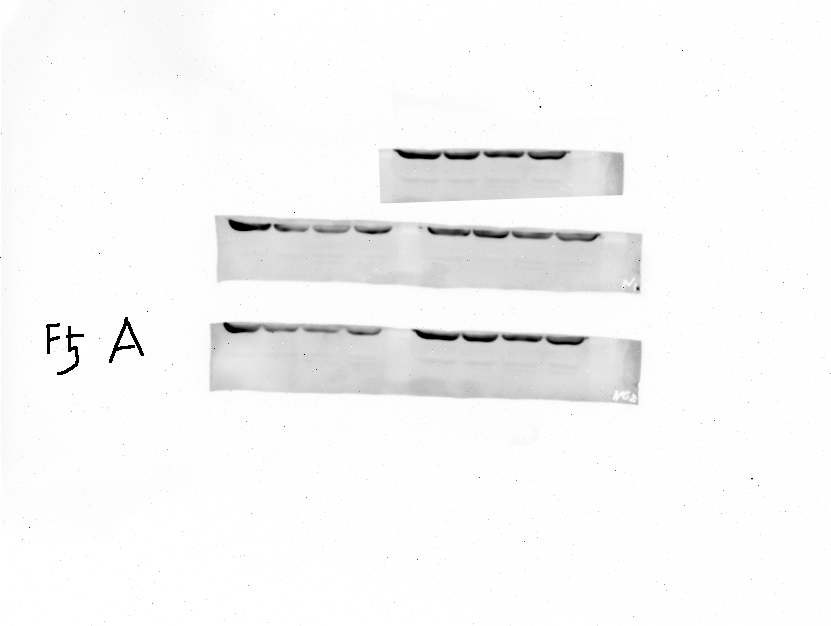



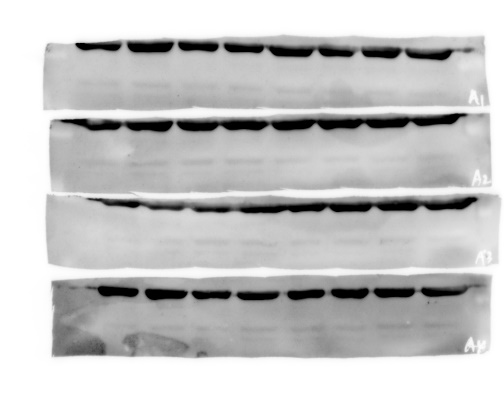

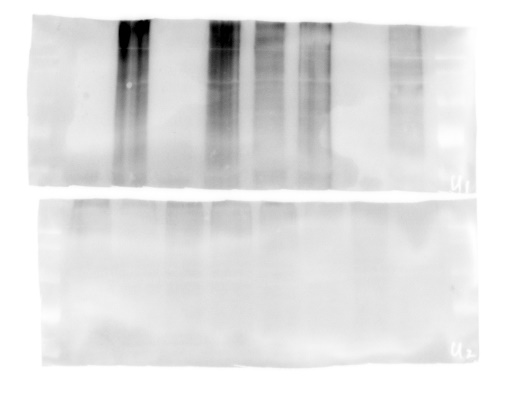




Figure 6

Supplement: Supplementary file 8 — Original Data File [file 41419_2023_6163_MOESM8_ESM.docx]
