# Supplementary material for: IGF2BP2-meidated m6A modification of CSF2 reprograms MSC to promote gastric cancer progression
Source: Cell Death Dis. 2023 Oct 21;14(10):693. doi: 10.1038/s41419-023-06163-7 (PMC10590395; doi:10.1038/s41419-023-06163-7)
Supplement: Supplementary file 7 — Supplementary Table 1 [file 41419_2023_6163_MOESM7_ESM.docx]

**Supplementary Table 1**

Specific primers for target mRNAs.

| Primers or oligonucleotides | Sequence (5' to 3') |
| --- | --- |
| H-β-actin-F | CACGAAACTACCTTCAACTCC |
| H-β-actin-R | CATACTCCTGCTTGCTGATC |
| H-CSF2-F | TCCTGAACCTGAGTAGAGACAC |
| H-CSF2-R | TGCTGCTTGTAGTGGCTGG |
| H-FAP-F | ATAGCAGTGGCTCCAGTCTC |
| H-FAP-R | GATAAGCCGTGGTTCTGGTC |
| H-α-SMA-F | CTGACTGAGCGTGGCTATTC |
| H-α-SMA-R | CCACCGATCCAGACAGAGTA |
| H-IGF2BP1-F | GCTCTTTGGGGACAGGAAGC |
| H-IGF2BP1-R | GGAGCTCACCTCTTCATCCG |
| H-IGF2BP2-F | AGCTAAGCGGGCATCAGTTTG |
| H-IGF2BP2-R | CCGCAGCGGGAAATCAATCT |
| H-IGF2BP3-F | TATATCGGAAACCTCAGCGAGA |
| H-IGF2BP3-R | GGACCGAGTGCTCAACTTCT |
| H-Notch1-F | AATGTGGATGCCGCAGTTG |
| H-Notch1-R | ATCCGTGATGTCCCGGTTG |
| H-Notch2-F | CCTGGGCTATACTGGGAGCTACTG |
| H-Notch2-R | ACACCCTGATAGCCTGGGACAC |
| H-Notch3-F | CTACGACTGTGTCTGCCAACCTG |
| H-Notch3-R | GCTGGAAGCACACTCATTGATCTC |
| H-Notch4-F | TGTTTGATGGCTACGACTGTGAGA |
| H-Notch4-R | AGTGCCCGTTGTGGAAGTGA |
| H-Jagged1-F | CATCGTGCTGCCTTTCAGT |
| H-Jagged1-R | GTTTTGTTGCCATTCTGGTCAC |
| H-Jagged2-F | TACTCGGGCAGGAACTGTG |
| H-Jagged2-R | TAGCCGCCAATCAGGTTTT |
| H-DLL1-F | GCAGGAGTTCGTCAACAAGAAG |
| H-DLL1-R | GCGAGGTCATCAGGAGAATC |
| H-DLL3-F | ATGGTCTCCCCACGGATGT |
| H-DLL3-R | CCTAACTCCTCTCTCCAGGTTTC |
| H-DLL4-F | ACTGCGAGAAGAAAGTGGACAG |
| H-DLL4-R | TGTGGAGAGGTCGGTGTAGC |
| H-HES1-F | ACTGATTTTGGATGCTCTGAAGA |
| H-HES1-R | GTATTAACGCCCTCGCACGT |
| H-N-cadherin-F | AGTCAACTGCAACCGTGTCT |
| H-N-cadherin-R | AGCGTTCCTGTTCCACTCAT |
| H-E-cadherin-F | CGCATTGCCACATACACTCT |
| H-E-cadherin-R | TTGGCTGAGGATGGTGTAAG |
| H-BTG2-F | CATCATCAGCAGGGTGGC |
| H-BTG2-R | CCCAATGCGGTAGGACAC |
| H-B3GALT2-F | GGAAGAGTCAACAGTGCTACAA |
| H-B3GALT2-R | AGTCTGCCTCCTGCGATTG |
| H-SVEP1-F | GAGACCCCAGACCTATTGC |
| H-SVEP1-R | ATATTCCCCTGCCAAATCCC |
| H-FOXC1-F | TGAACGGGAATAGTAGCTGTCA |
| H-FOXC1-R | GGACGTGCGGTACAGAGAC |
| H-HLA-DMA-F | AGTCTCTTTTTCCCCCTACAC |
| H-HLA-DMA-R | ATCTATC-CCTTTTTGCCCCCA |
| H-EPOP-F | CCTTCAGCCTCCTCAACT |
| H-EPOP-R | ATCTATGTCATCAGCCTTCTC |
| H-SEC31B-F | GGAGAGGAGGCTGAGATT |
| H-SEC31B-R | TGTGGAGGCTGGATAGAG |
| H-CCPG1-F | AAGCGTCAGCAGTTAGGCAGAAAG |
| H-CCPG1-R | TCGGTAGTGGTCCAACACCTCTC |

The sequences and modifications of the oligonucleotides.

| Oligonucleotides | Sequences (5'-3') | Modifications |
| --- | --- | --- |
| si-CSF2 | CUGAACCUGAGUAGAGACATT  UGUCUCUACUCAGGUUCAGTT | 2’Ome |
| si-IGF2BP2 | GCGAAAGGAUGGUCAUCAUTT  AUGAUGACCAUCCUUUCGCTT | 2’Ome |
| si-Notch1 | GCAACAGCUCCUUCCACUUTT  AAGUGGAAGGAGCUGUUGCTT | 2’Ome |
| si-METTL3 | GCUCAACAUACCCGUACUATT UAGUACGGGUAUGUUGAGCTT | 2’Ome |
| Scr of si-RNA | UUCUCCGAACGUGUCACGUTT  ACGUGACACGUUCGGAGAATT |  |
